# Supplementary figures and images for: SLC35G3 is a UDP-N-acetylglucosamine transporter for sperm glycoprotein formation and underpins male fertility in mice
Source: eLife. 2025 Nov 12;14:RP107494. doi: 10.7554/eLife.107494 (PMC12611266; doi:10.7554/eLife.107494)

Fig1B  
TOP

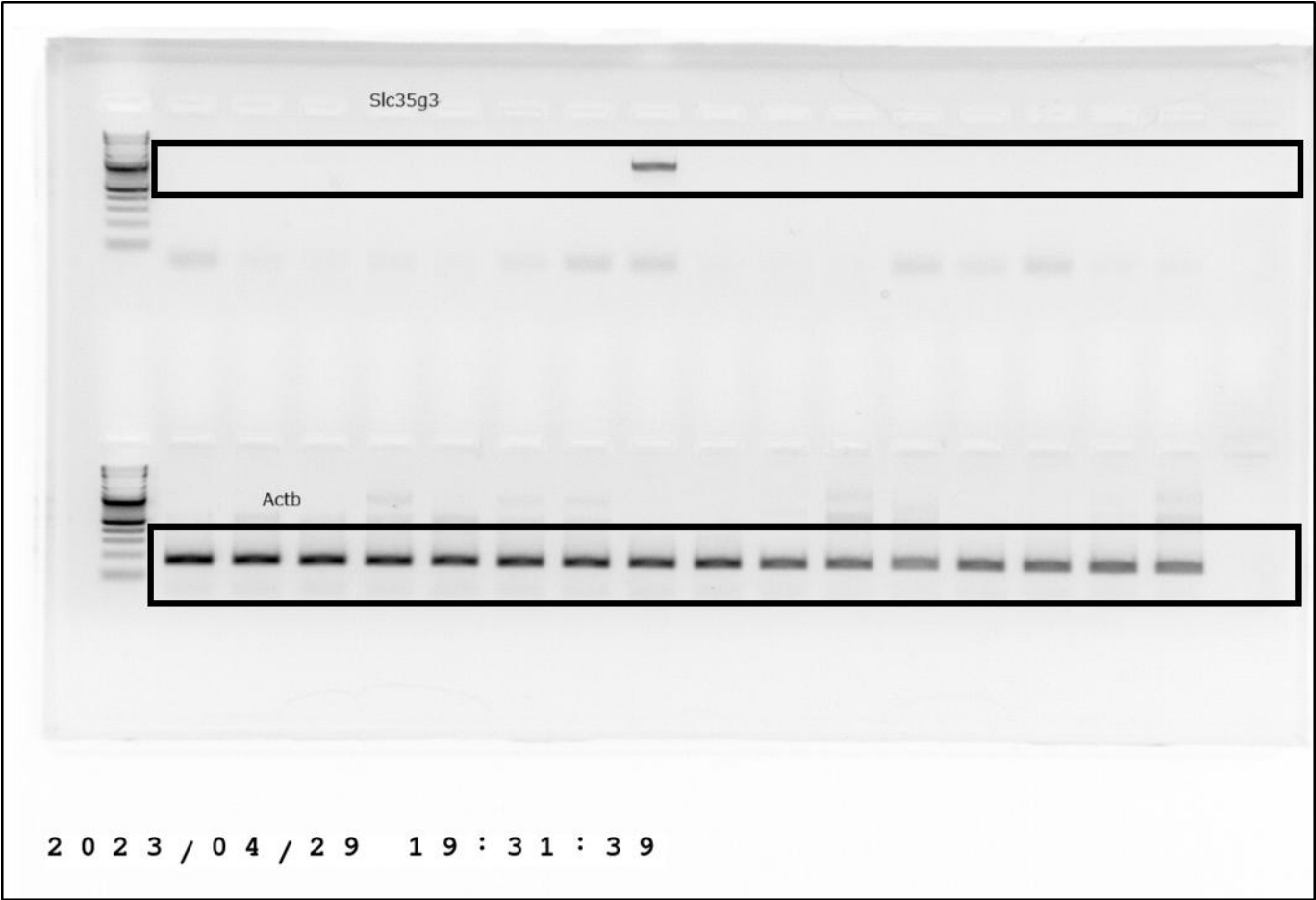

Fig1B  
BOTTOM

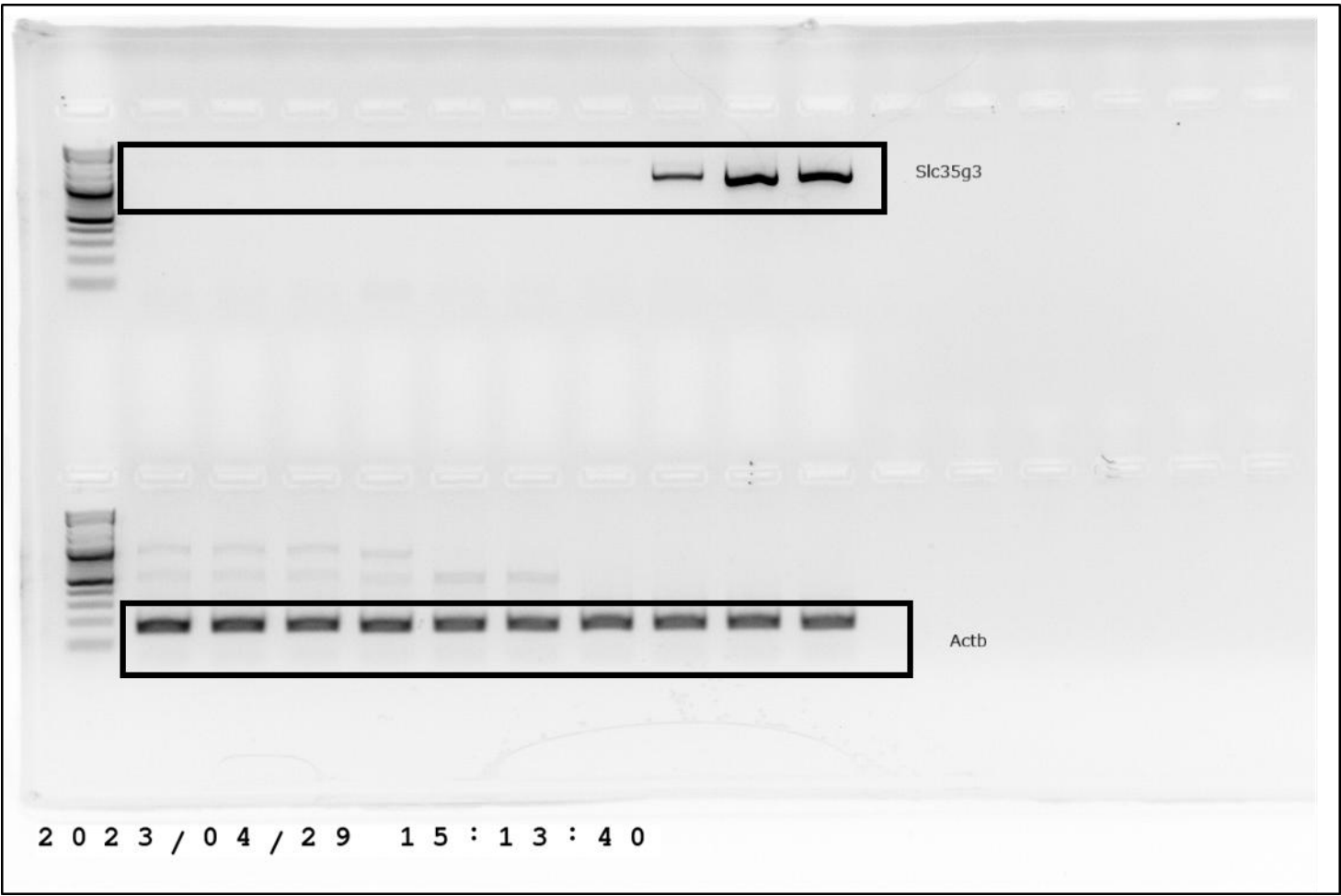

Supplement: Figure 1—source data 1. [file elife-107494-fig1-data1.pdf]

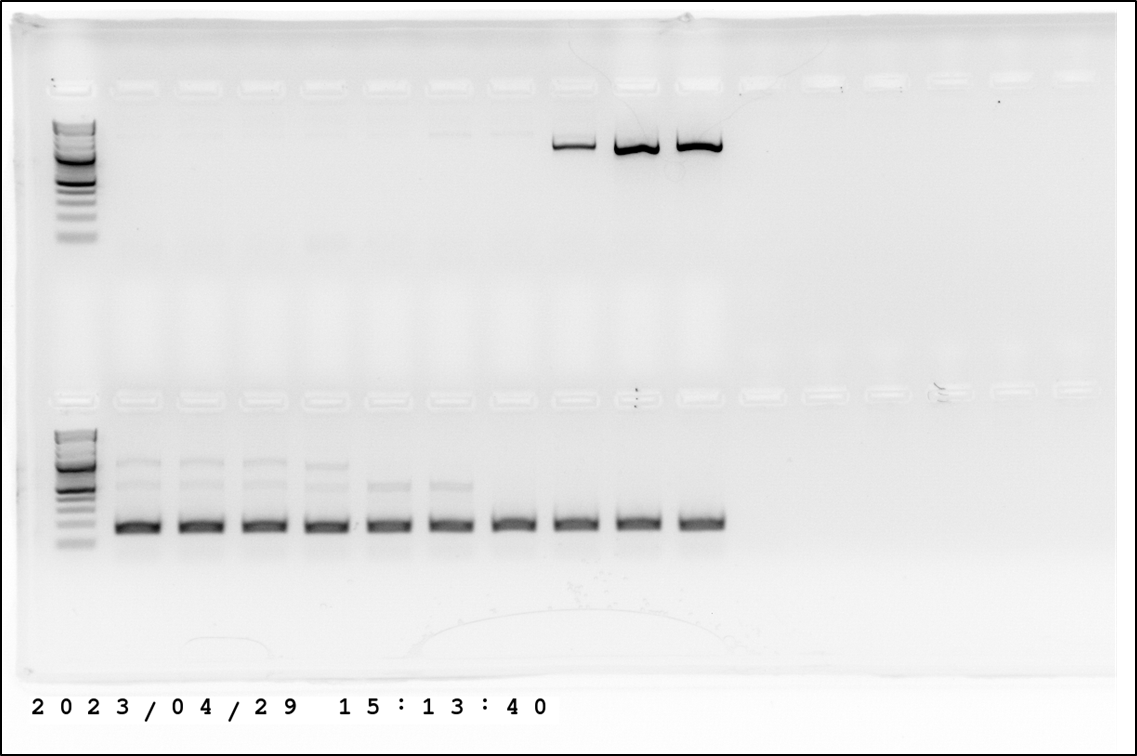

Supplement: Figure 1—source data 2. [file elife-107494-fig1-data2.zip › Figure1-sourcedata2/fig1b bot.tif]

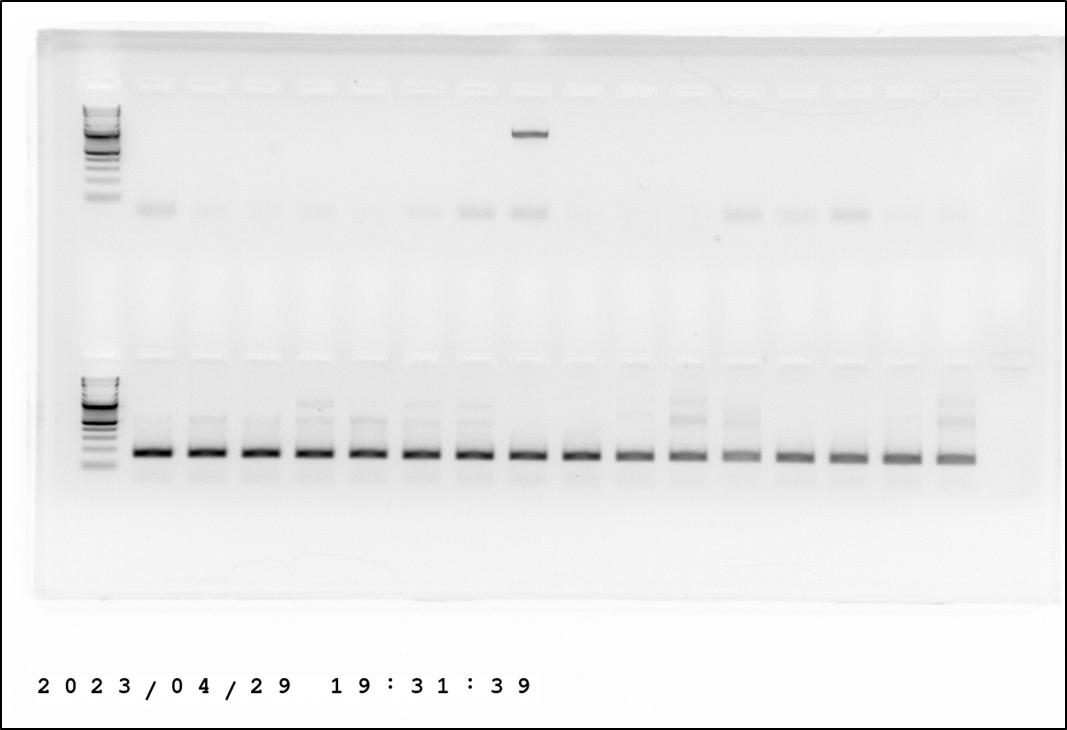

Supplement: Figure 1—source data 2. [file elife-107494-fig1-data2.zip › Figure1-sourcedata2/fig1b top.tif]

mCherry

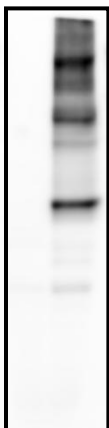

BASIGIN

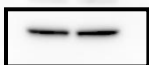

Supplement: Figure 1—figure supplement 2—source data 1. [file elife-107494-fig1-figsupp2-data1.zip › Figure1-figuresupplement2-sourcedata1.pdf]

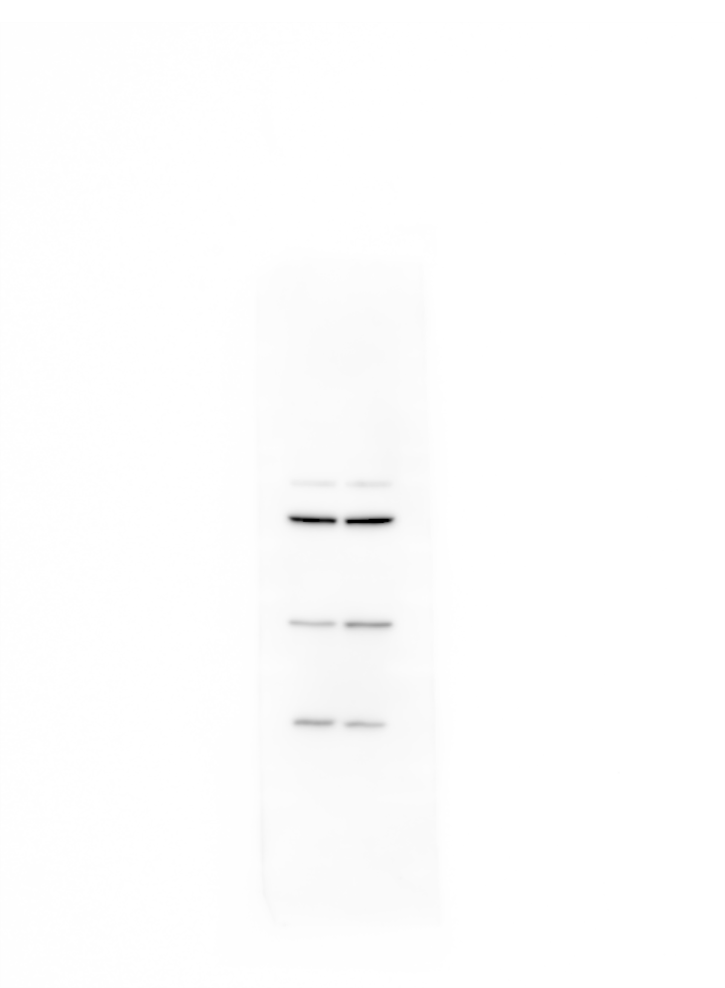

Supplement: Figure 1—figure supplement 2—source data 2. [file elife-107494-fig1-figsupp2-data2.zip › Figure1-figuresupplement2-sourcedata2/BASIGIN.tif]

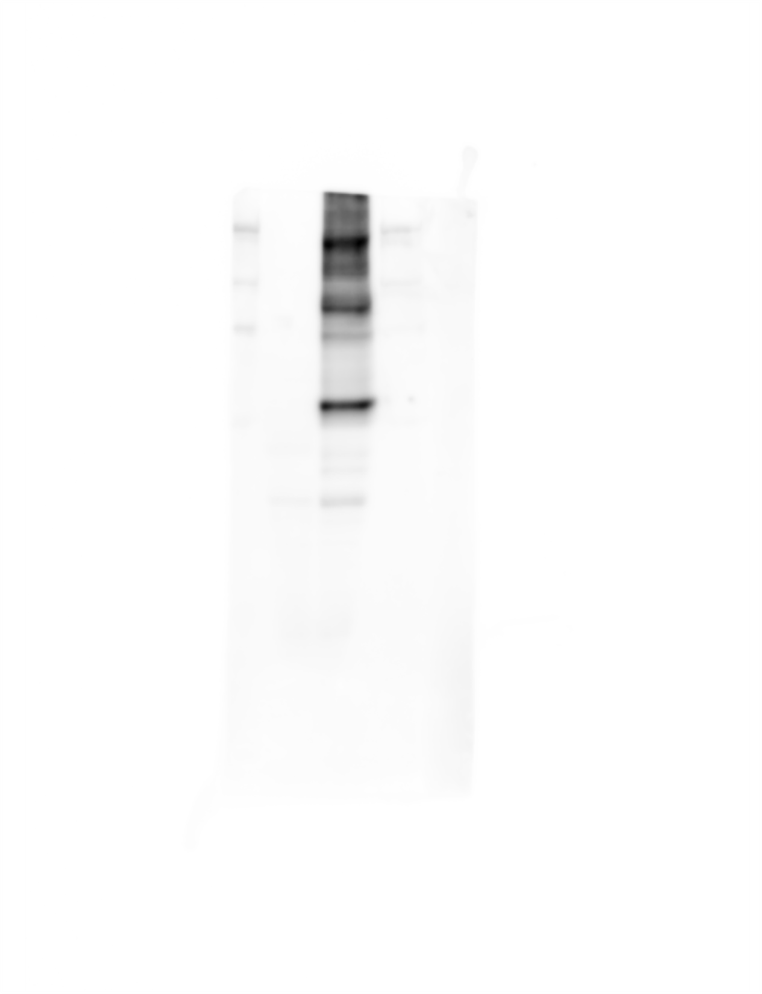

Supplement: Figure 1—figure supplement 2—source data 2. [file elife-107494-fig1-figsupp2-data2.zip › Figure1-figuresupplement2-sourcedata2/mCherry.tif]

Figure2B

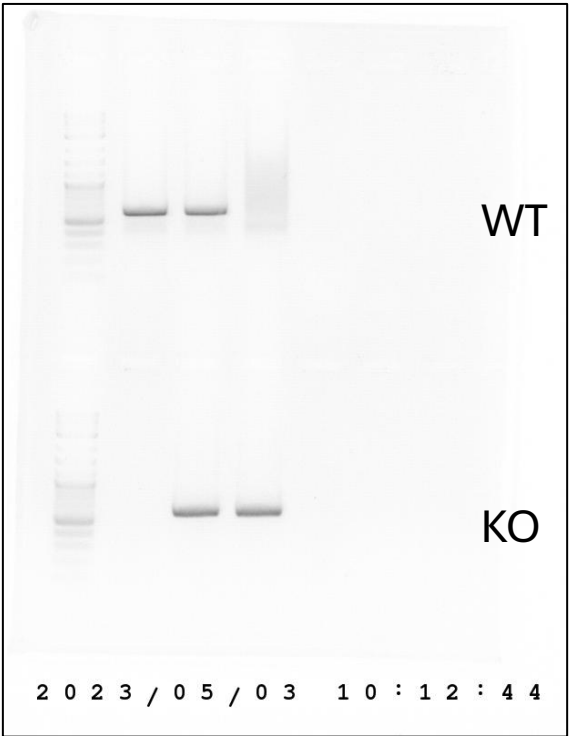

Figure2C

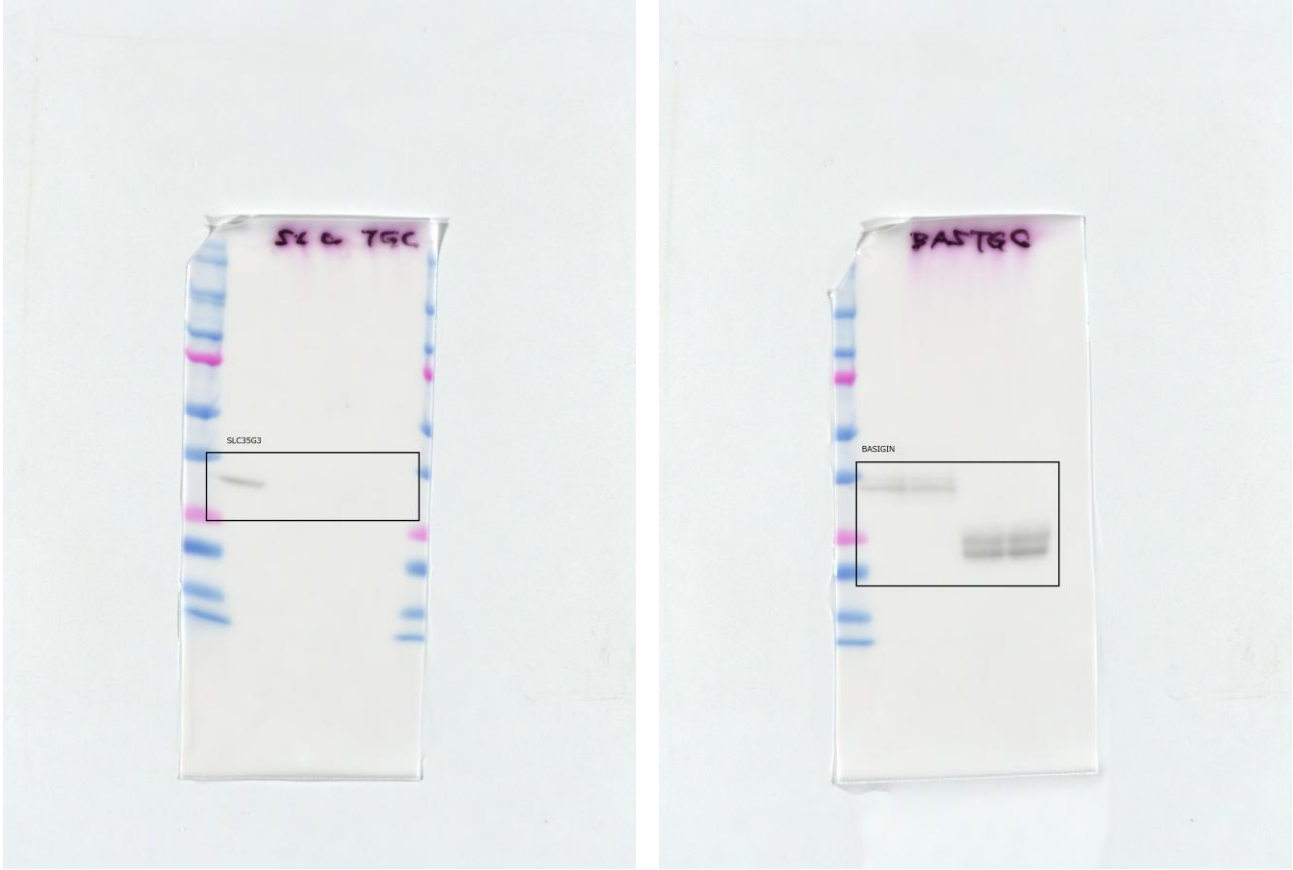

Supplement: Figure 2—source data 1. [file elife-107494-fig2-data1.pdf]

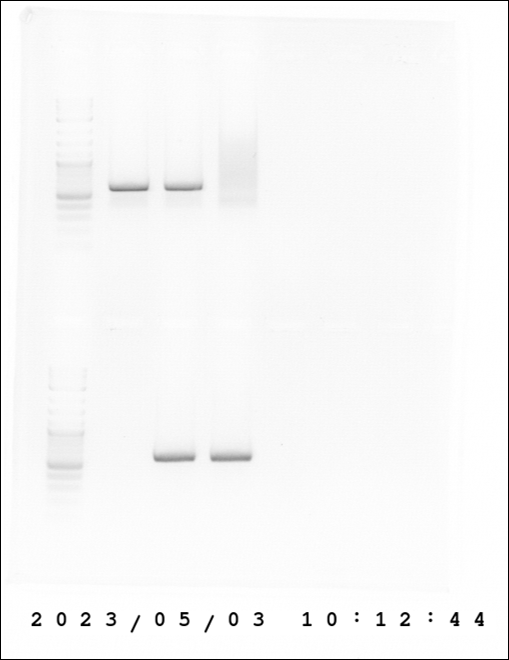

Supplement: Figure 2—source data 2. [file elife-107494-fig2-data2.zip › Figure2-sourcedata2/Fig2-sourcedata2-2B.tif]

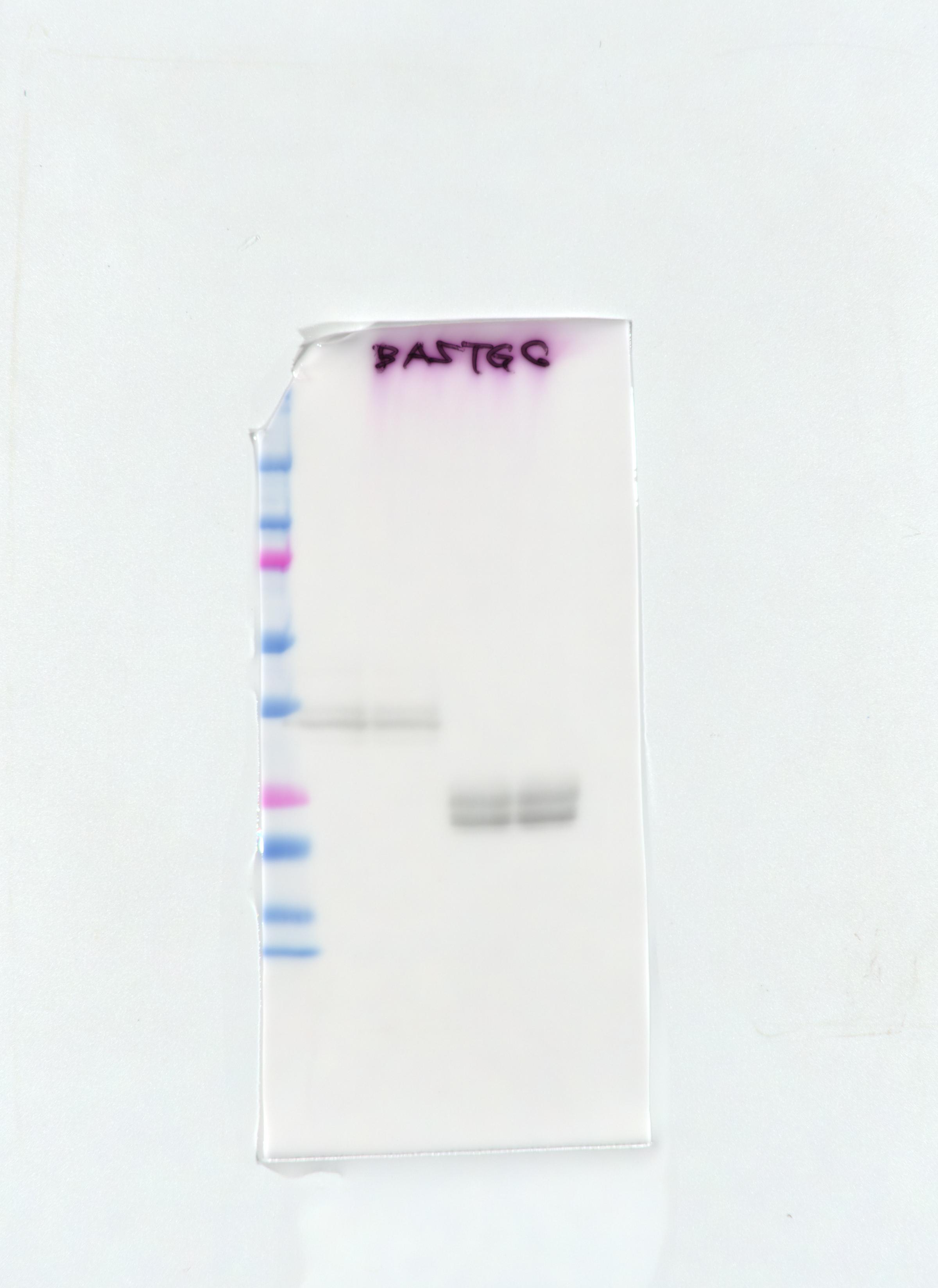

Supplement: Figure 2—source data 2. [file elife-107494-fig2-data2.zip › Figure2-sourcedata2/Fig2C-basigin.jpg]

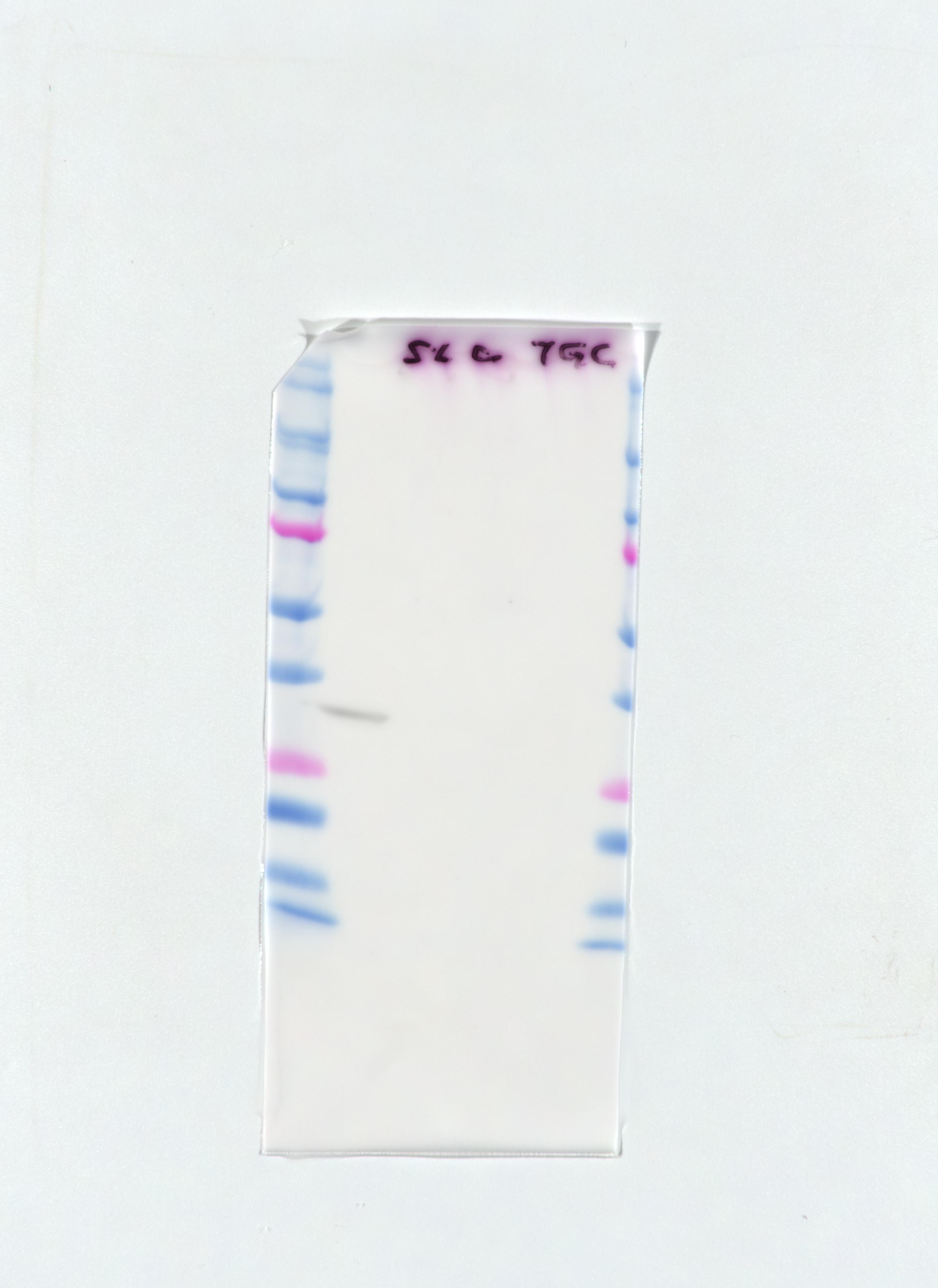

Supplement: Figure 2—source data 2. [file elife-107494-fig2-data2.zip › Figure2-sourcedata2/Fig2C-SLC35G3.jpg]

A

SPACA1

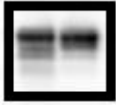

ZPBP1

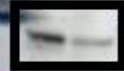

GOPC

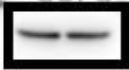

BASIGIN

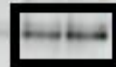

**B**

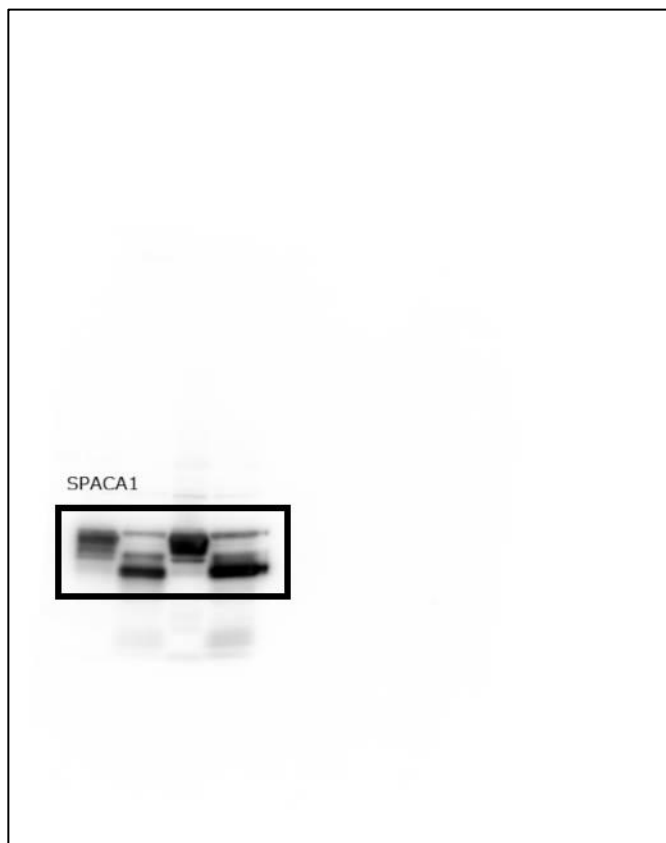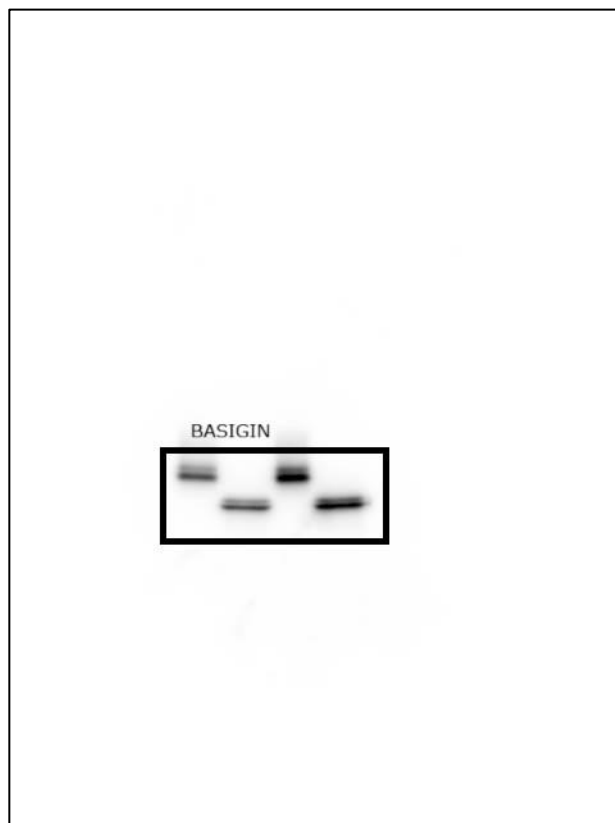

C

SPACA1

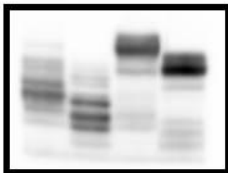

Bas

BASIGIN

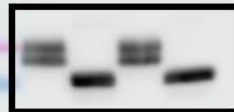

D

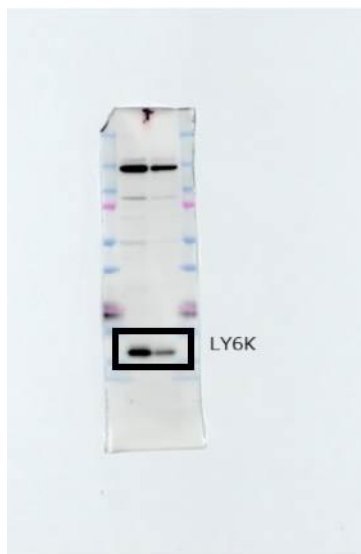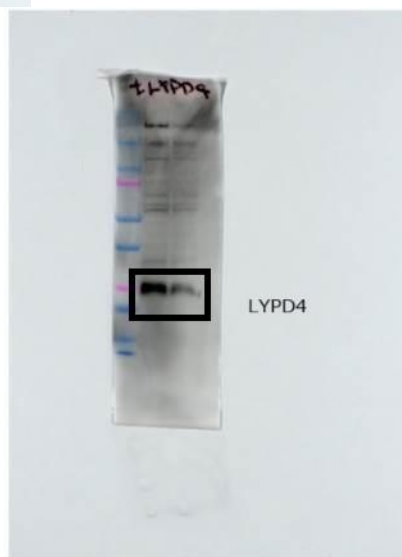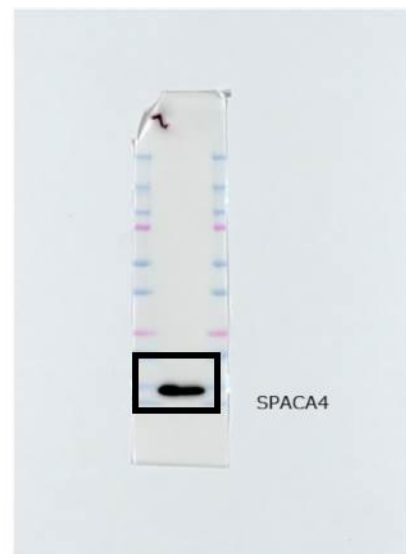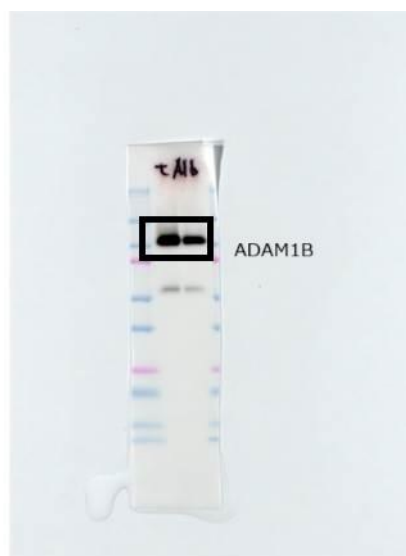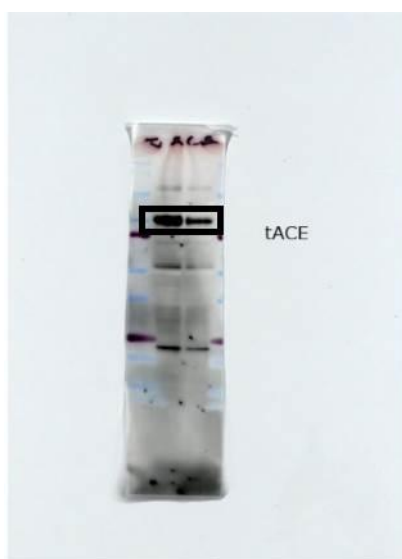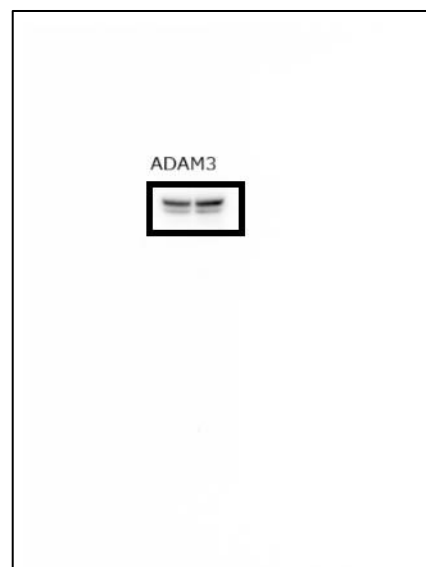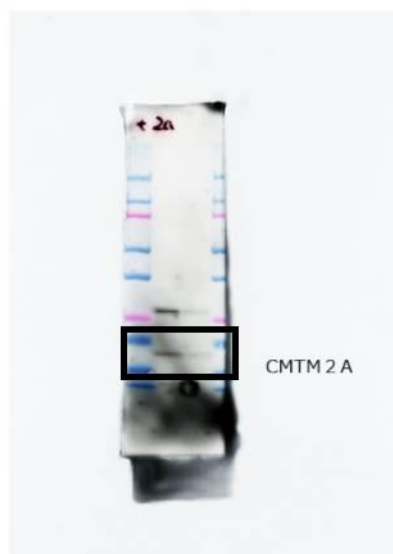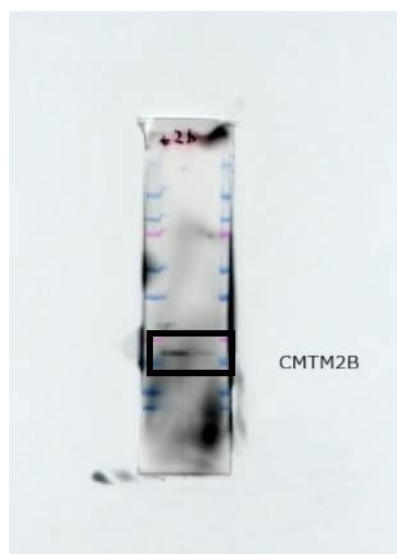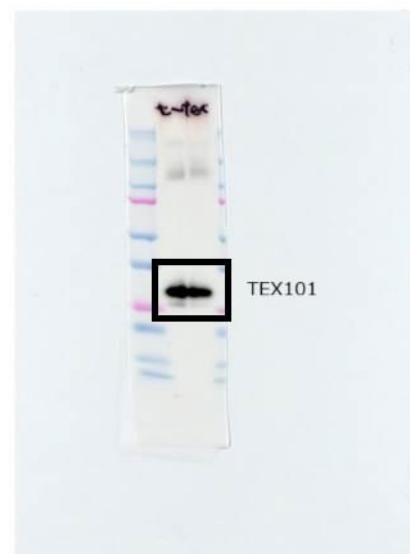

D

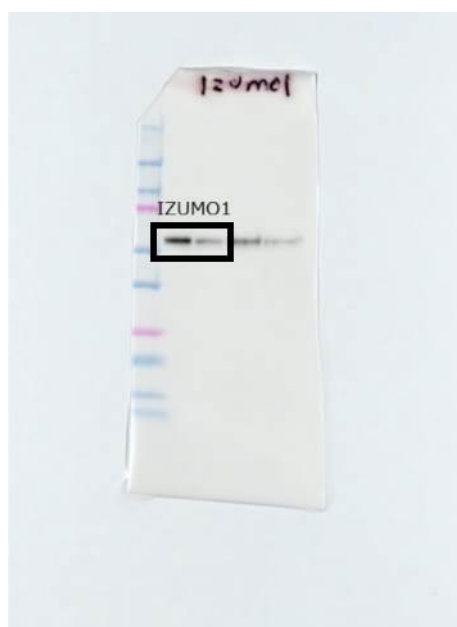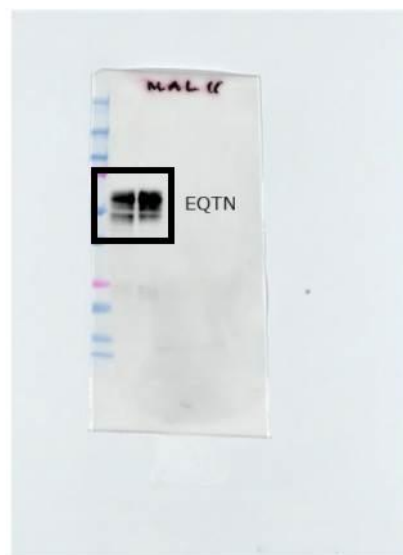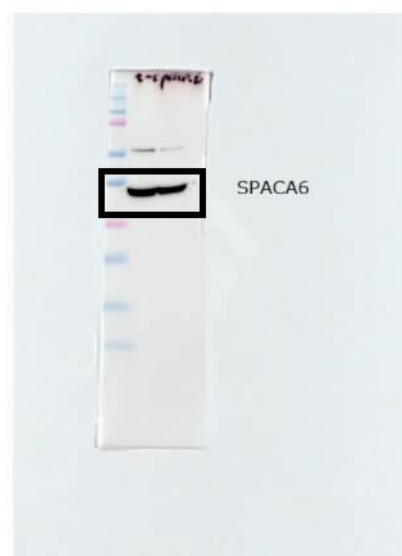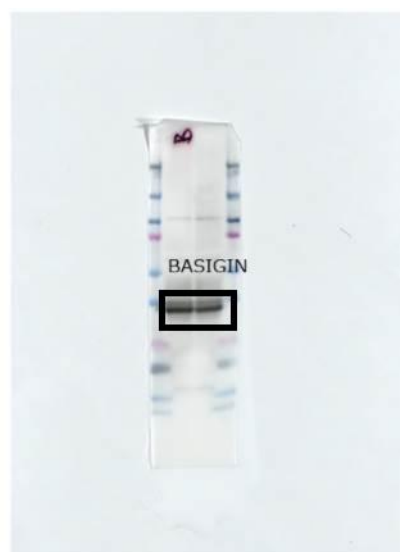

E

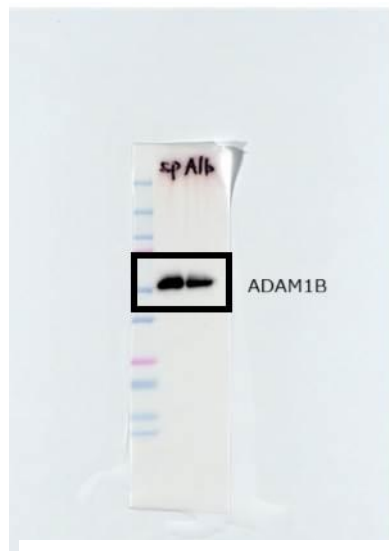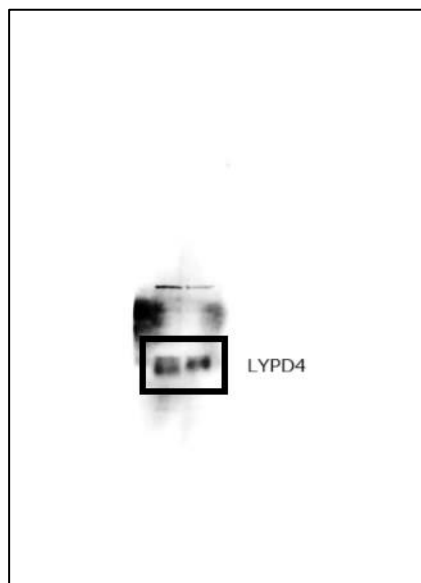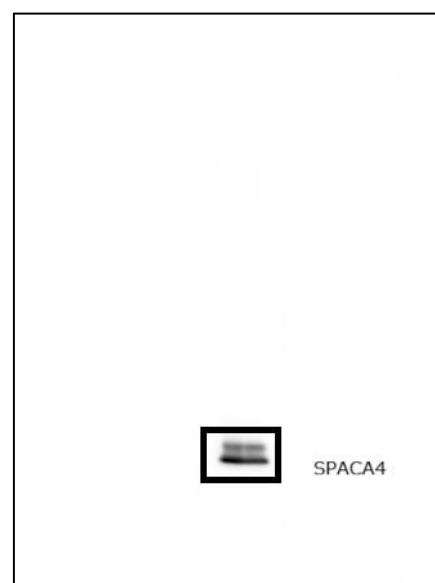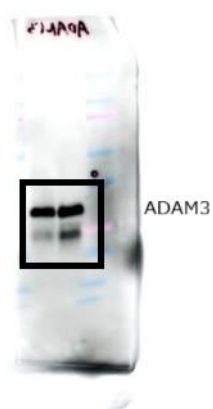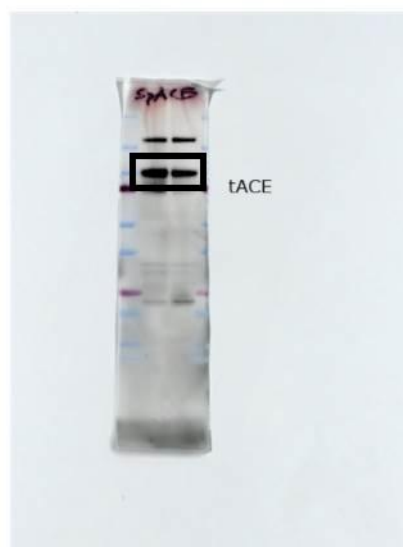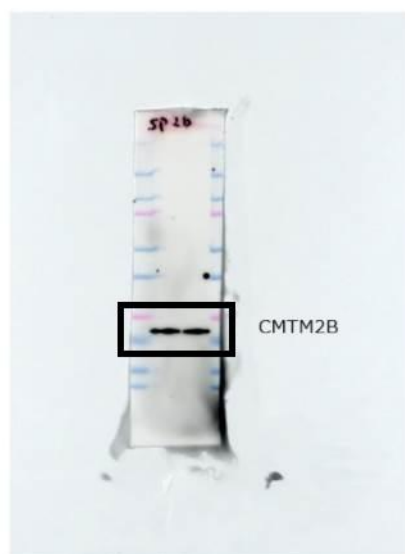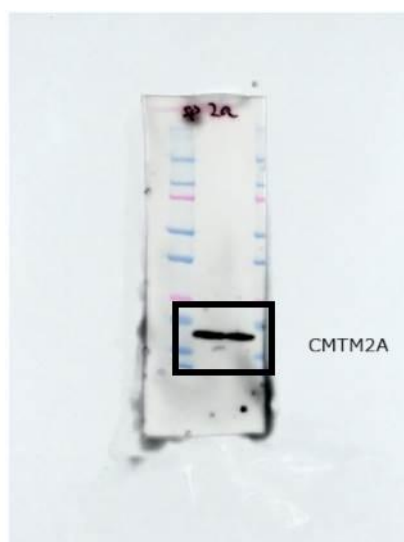

E

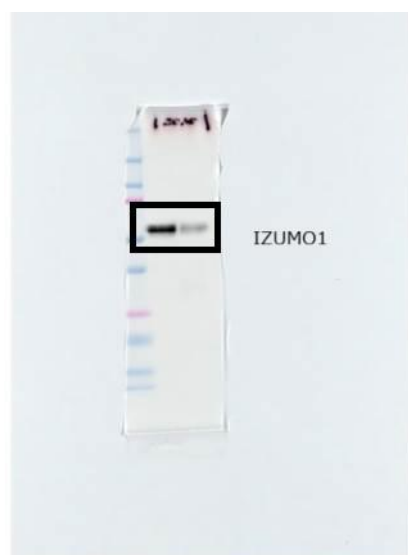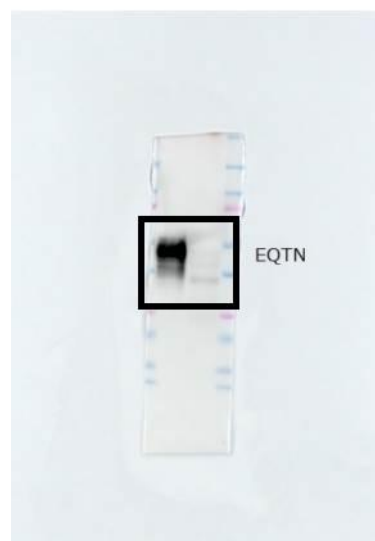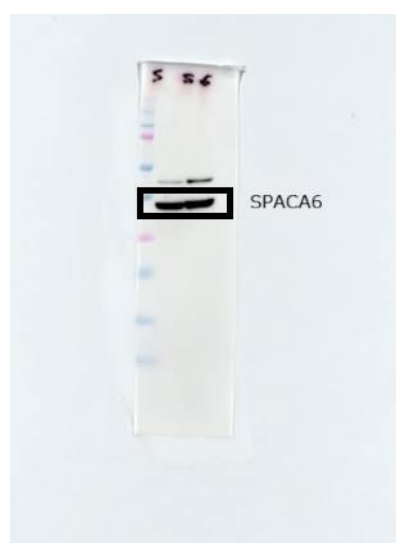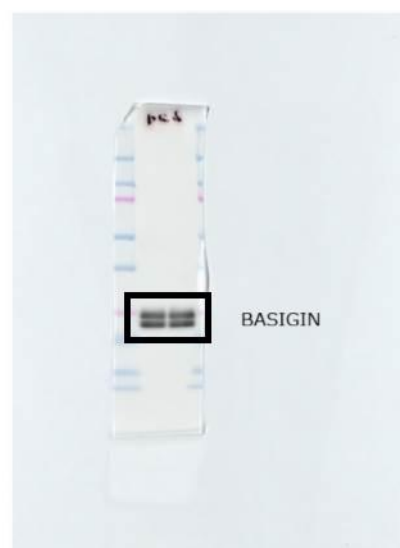

Supplement: Figure 6—source data 1. [file elife-107494-fig6-data1.pdf]

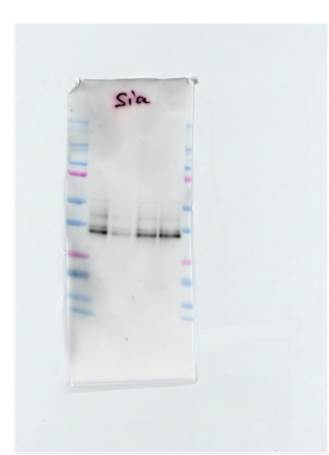

Supplement: Figure 6—source data 2. [file elife-107494-fig6-data2.zip › Figure6-source data2/f6a_BASIGIN.jpg]

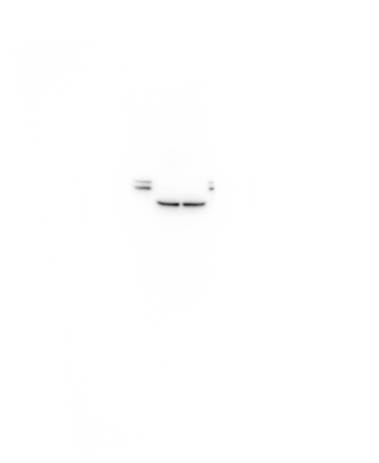

Supplement: Figure 6—source data 2. [file elife-107494-fig6-data2.zip › Figure6-source data2/f6a_GOPC.jpg]

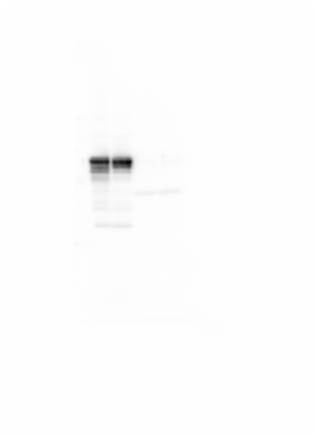

Supplement: Figure 6—source data 2. [file elife-107494-fig6-data2.zip › Figure6-source data2/f6a_spaca1.jpg]

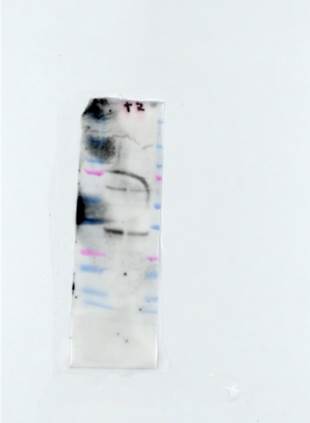

Supplement: Figure 6—source data 2. [file elife-107494-fig6-data2.zip › Figure6-source data2/f6a_zpbp1.jpg]

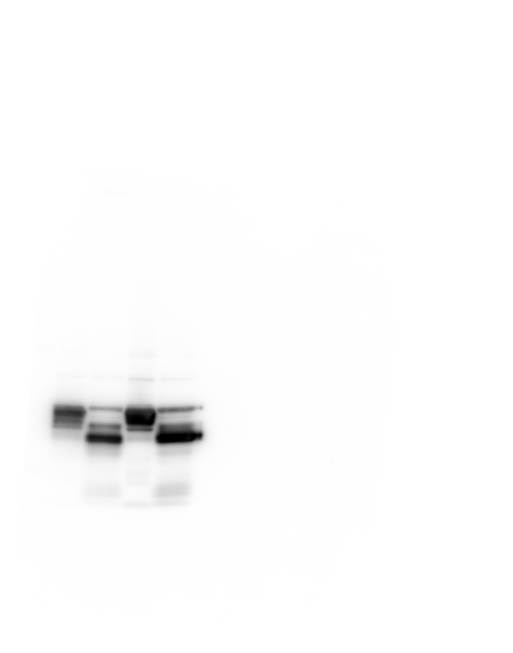

Supplement: Figure 6—source data 2. [file elife-107494-fig6-data2.zip › Figure6-source data2/f6b-spaca1.jpg]

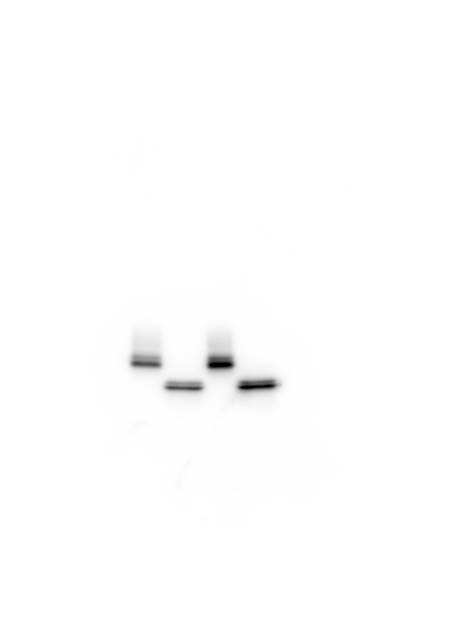

Supplement: Figure 6—source data 2. [file elife-107494-fig6-data2.zip › Figure6-source data2/f6b_BASIGIN.jpg]

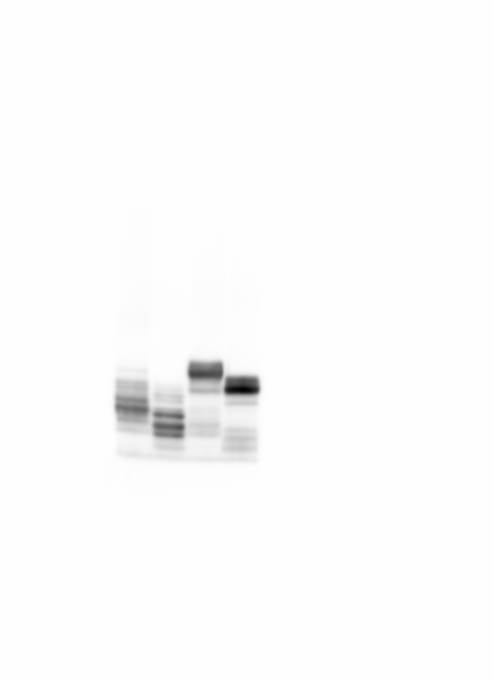

Supplement: Figure 6—source data 2. [file elife-107494-fig6-data2.zip › Figure6-source data2/f6c-spaca1.jpg]

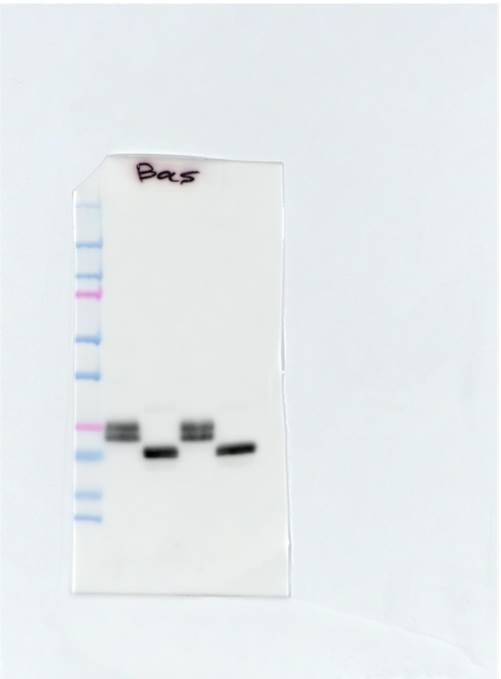

Supplement: Figure 6—source data 2. [file elife-107494-fig6-data2.zip › Figure6-source data2/f6c_BASIGIN.jpg]

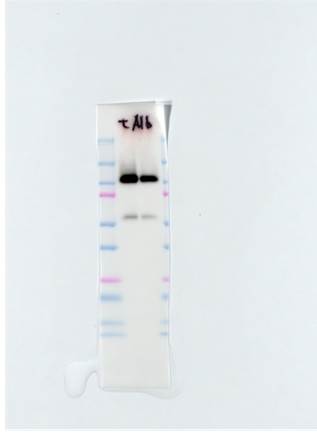

Supplement: Figure 6—source data 2. [file elife-107494-fig6-data2.zip › Figure6-source data2/f6d-ADAM1B.jpg]

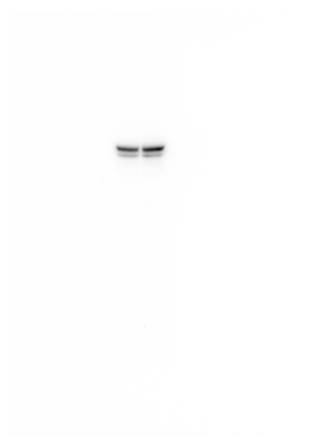

Supplement: Figure 6—source data 2. [file elife-107494-fig6-data2.zip › Figure6-source data2/f6d-ADAM3.jpg]

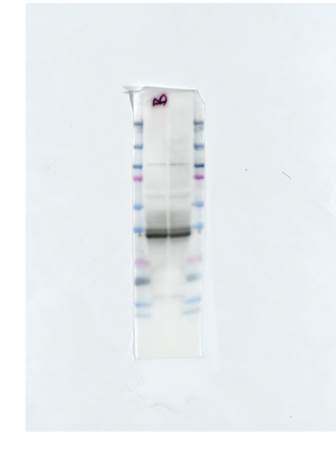

Supplement: Figure 6—source data 2. [file elife-107494-fig6-data2.zip › Figure6-source data2/f6d-BASIGIN.jpg]

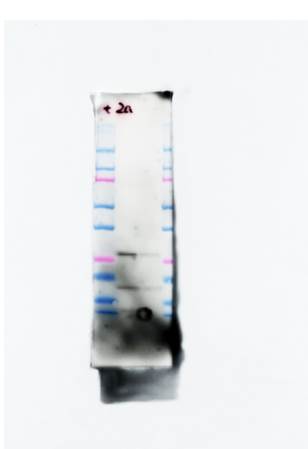

Supplement: Figure 6—source data 2. [file elife-107494-fig6-data2.zip › Figure6-source data2/f6d-CMTM2A.jpg]

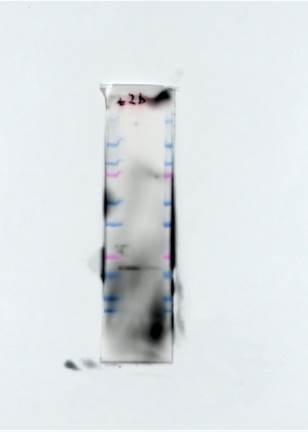

Supplement: Figure 6—source data 2. [file elife-107494-fig6-data2.zip › Figure6-source data2/f6d-CMTM2B.jpg]

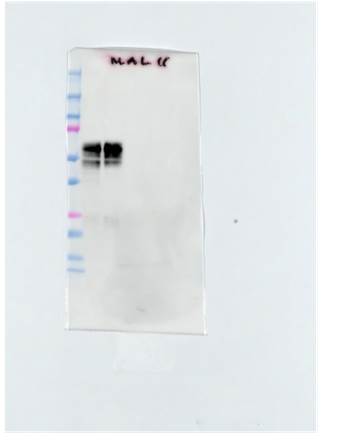

Supplement: Figure 6—source data 2. [file elife-107494-fig6-data2.zip › Figure6-source data2/f6d-EQTN.jpg]

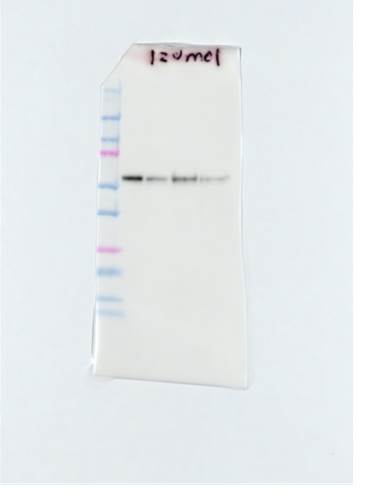

Supplement: Figure 6—source data 2. [file elife-107494-fig6-data2.zip › Figure6-source data2/f6d-IZUMO1.jpg]

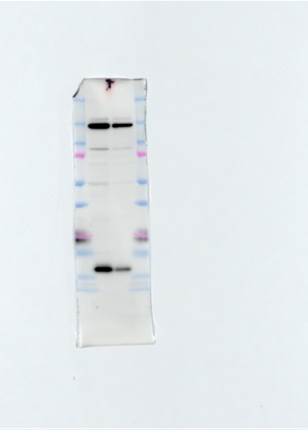

Supplement: Figure 6—source data 2. [file elife-107494-fig6-data2.zip › Figure6-source data2/f6d-Ly6k.jpg]

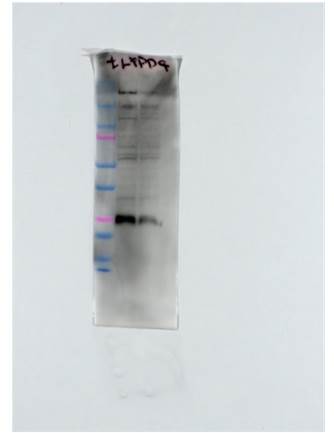

Supplement: Figure 6—source data 2. [file elife-107494-fig6-data2.zip › Figure6-source data2/f6d-LYPD4.jpg]

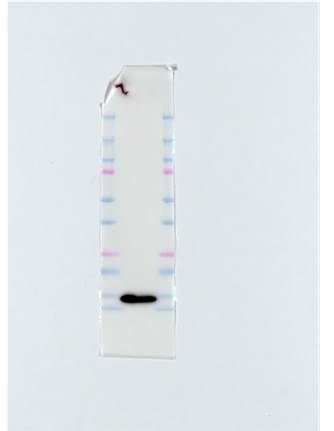

Supplement: Figure 6—source data 2. [file elife-107494-fig6-data2.zip › Figure6-source data2/f6d-SPACA4.jpg]

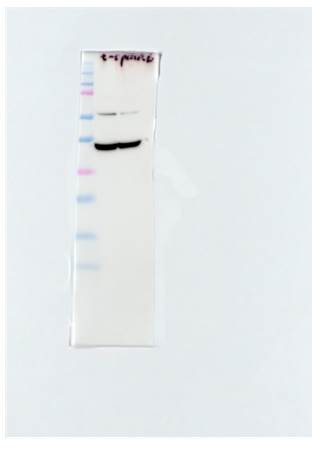

Supplement: Figure 6—source data 2. [file elife-107494-fig6-data2.zip › Figure6-source data2/f6d-SPACA6.jpg]

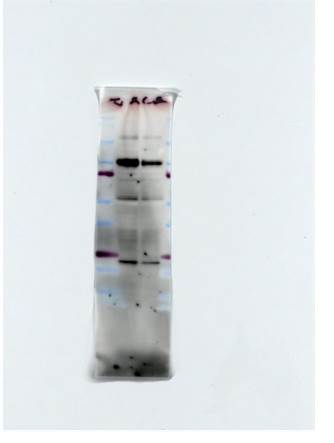

Supplement: Figure 6—source data 2. [file elife-107494-fig6-data2.zip › Figure6-source data2/f6d-tACE.jpg]

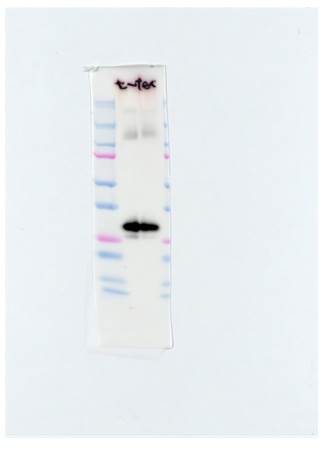

Supplement: Figure 6—source data 2. [file elife-107494-fig6-data2.zip › Figure6-source data2/f6d-TEX101.jpg]

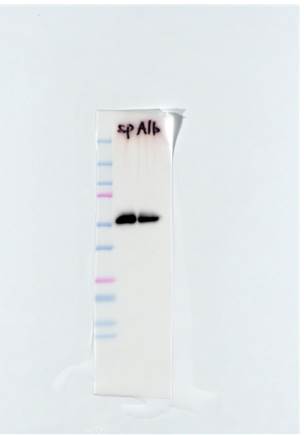

Supplement: Figure 6—source data 2. [file elife-107494-fig6-data2.zip › Figure6-source data2/f6e-ADAM1B.jpg]

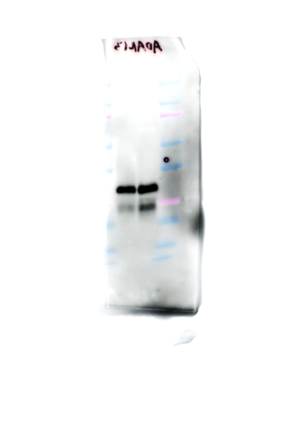

Supplement: Figure 6—source data 2. [file elife-107494-fig6-data2.zip › Figure6-source data2/f6e-ADAM3.jpg]

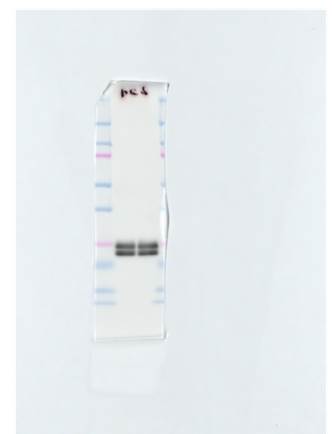

Supplement: Figure 6—source data 2. [file elife-107494-fig6-data2.zip › Figure6-source data2/f6e-BASIGIN.jpg]

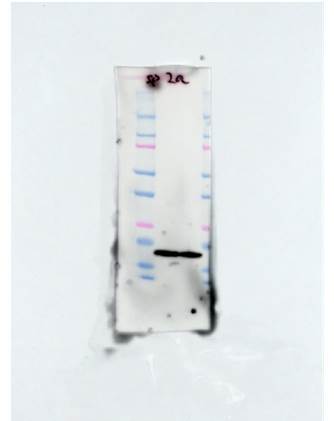

Supplement: Figure 6—source data 2. [file elife-107494-fig6-data2.zip › Figure6-source data2/f6e-CMTM2A.jpg]

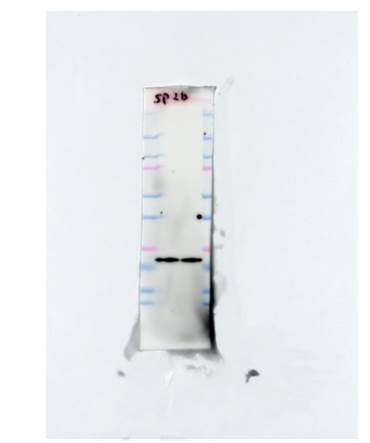

Supplement: Figure 6—source data 2. [file elife-107494-fig6-data2.zip › Figure6-source data2/f6e-CMTM2B.jpg]

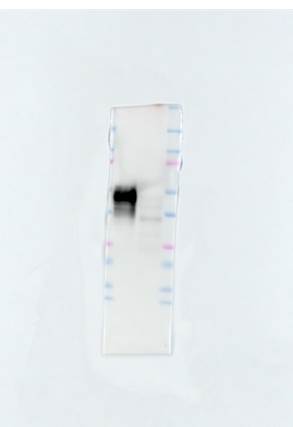

Supplement: Figure 6—source data 2. [file elife-107494-fig6-data2.zip › Figure6-source data2/f6e-EQTN.jpg]

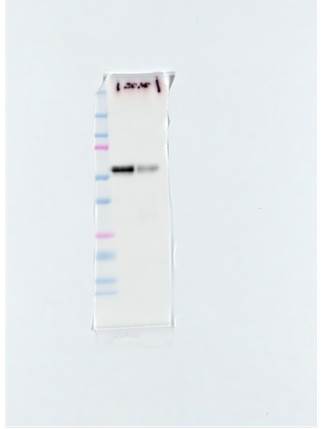

Supplement: Figure 6—source data 2. [file elife-107494-fig6-data2.zip › Figure6-source data2/f6e-IZUMO1.jpg]

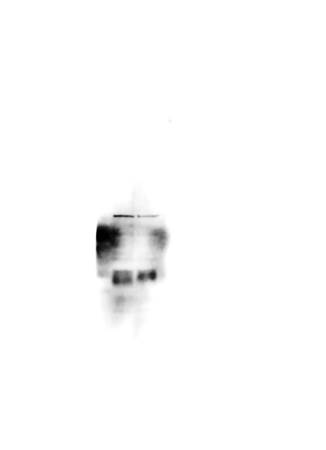

Supplement: Figure 6—source data 2. [file elife-107494-fig6-data2.zip › Figure6-source data2/f6e-LYPD4.jpg]

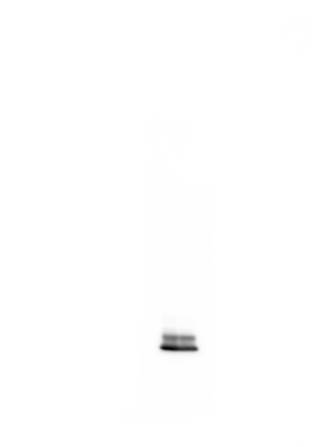

Supplement: Figure 6—source data 2. [file elife-107494-fig6-data2.zip › Figure6-source data2/f6e-SPACA4.jpg]

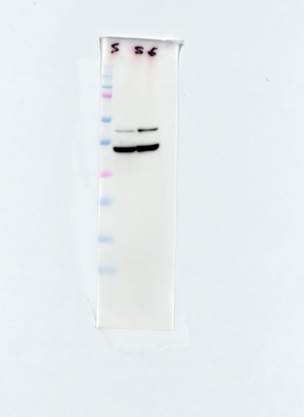

Supplement: Figure 6—source data 2. [file elife-107494-fig6-data2.zip › Figure6-source data2/f6e-SPACA6.jpg]

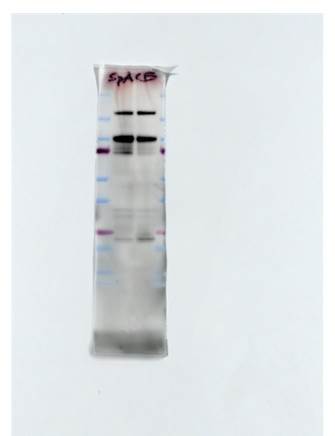

Supplement: Figure 6—source data 2. [file elife-107494-fig6-data2.zip › Figure6-source data2/f6e-tACE.jpg]

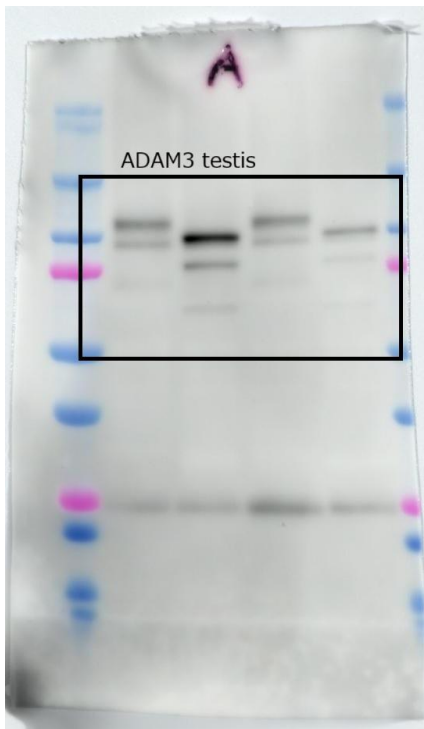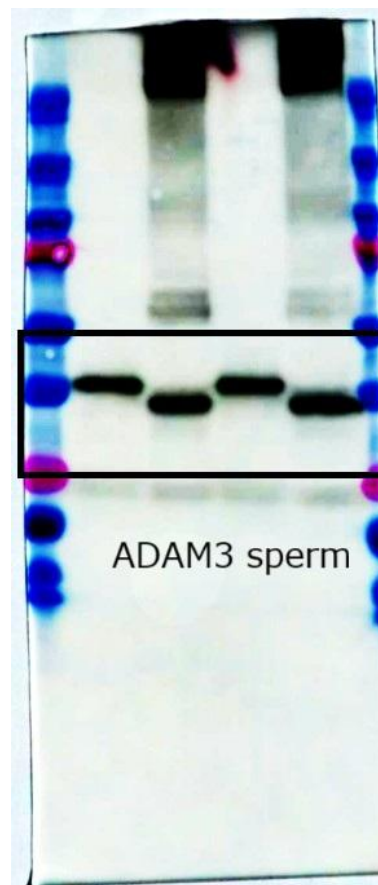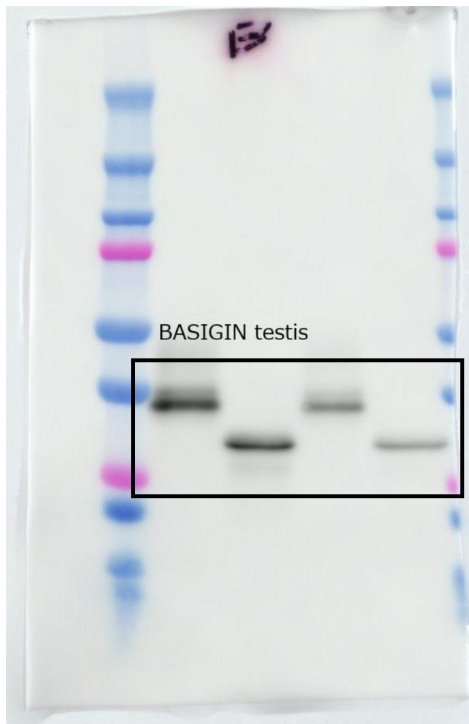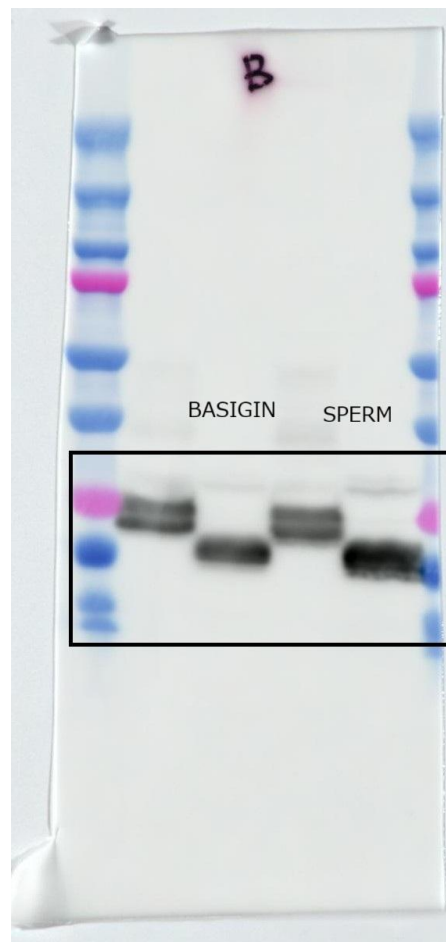

Supplement: Figure 6—figure supplement 1—source data 1. [file elife-107494-fig6-figsupp1-data1.pdf]

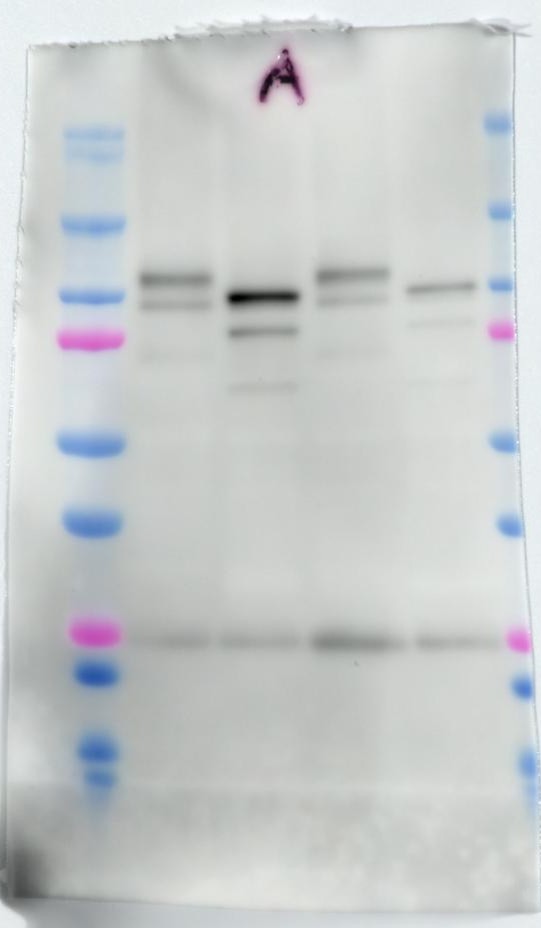

Supplement: Figure 6—figure supplement 1—source data 2. [file elife-107494-fig6-figsupp1-data2.zip › Figure6-supplement1-sourcedata2/Figure6-figure supplement1-source data 2 ADAM3testis.jpg]

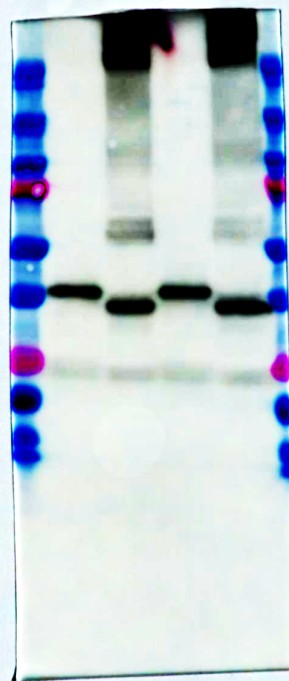

Supplement: Figure 6—figure supplement 1—source data 2. [file elife-107494-fig6-figsupp1-data2.zip › Figure6-supplement1-sourcedata2/Figure6-figure supplement1-source data 2_ADAM3sperm.jpg]

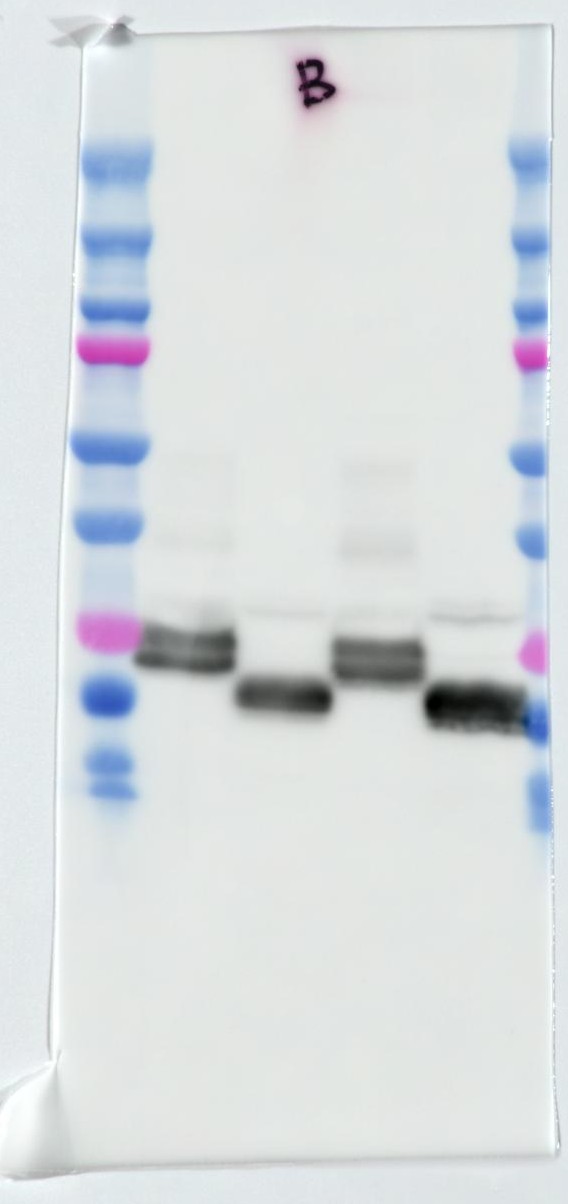

Supplement: Figure 6—figure supplement 1—source data 2. [file elife-107494-fig6-figsupp1-data2.zip › Figure6-supplement1-sourcedata2/Figure6-figure supplement1-source data 2_BASIGINsperm.jpg]

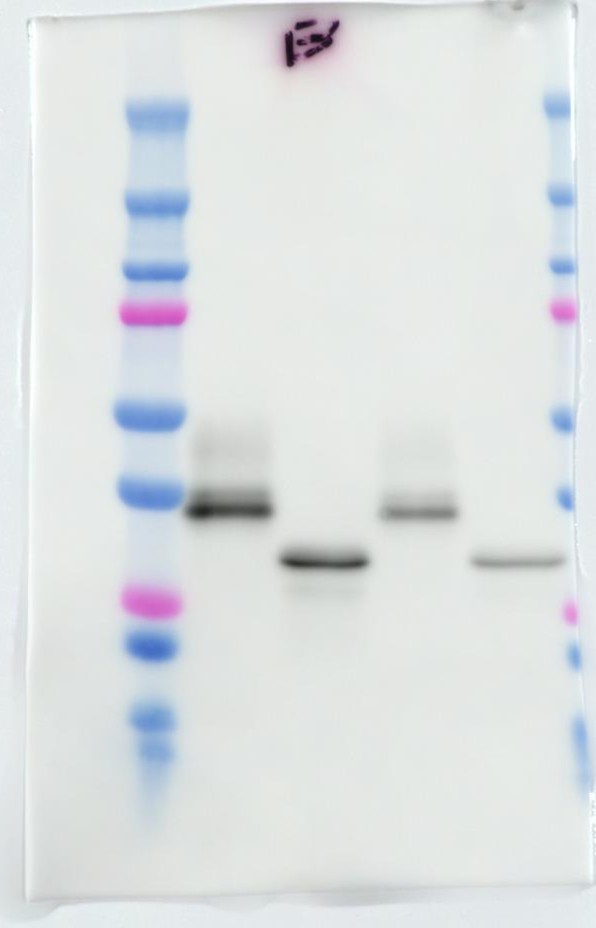

Supplement: Figure 6—figure supplement 1—source data 2. [file elife-107494-fig6-figsupp1-data2.zip › Figure6-supplement1-sourcedata2/Figure6-figure supplement1-source data 2_BASIGINtestis.jpg]

A

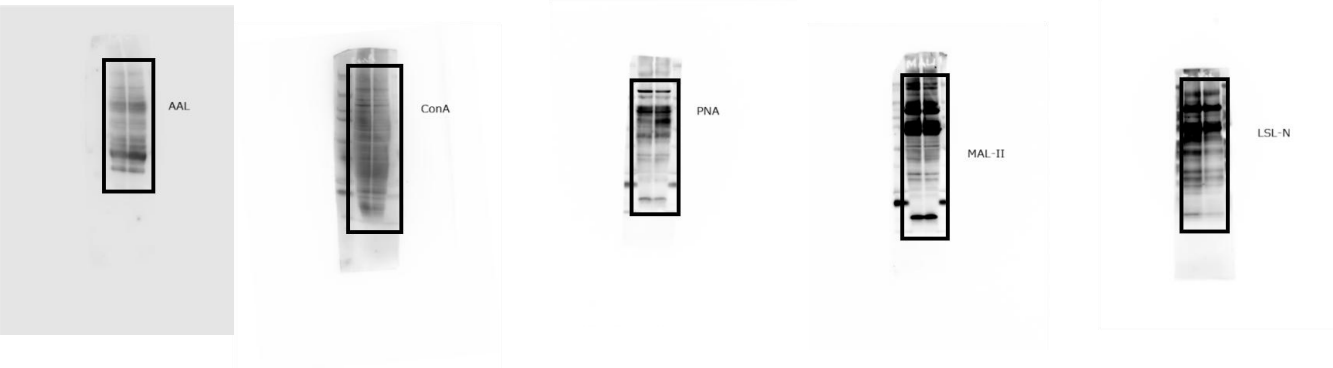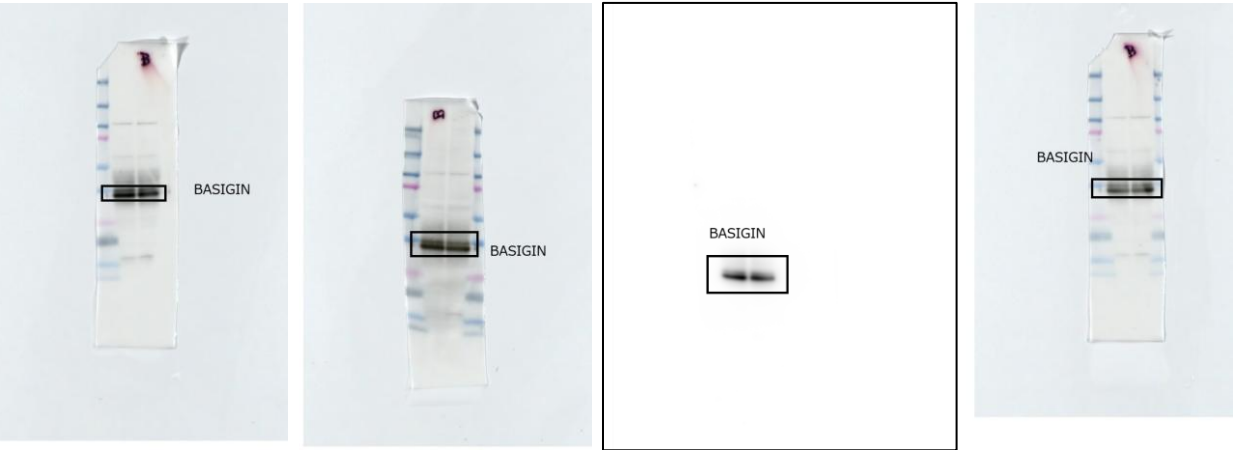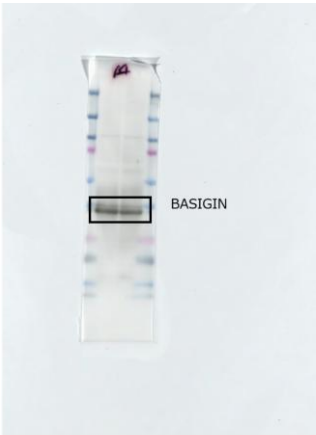

B

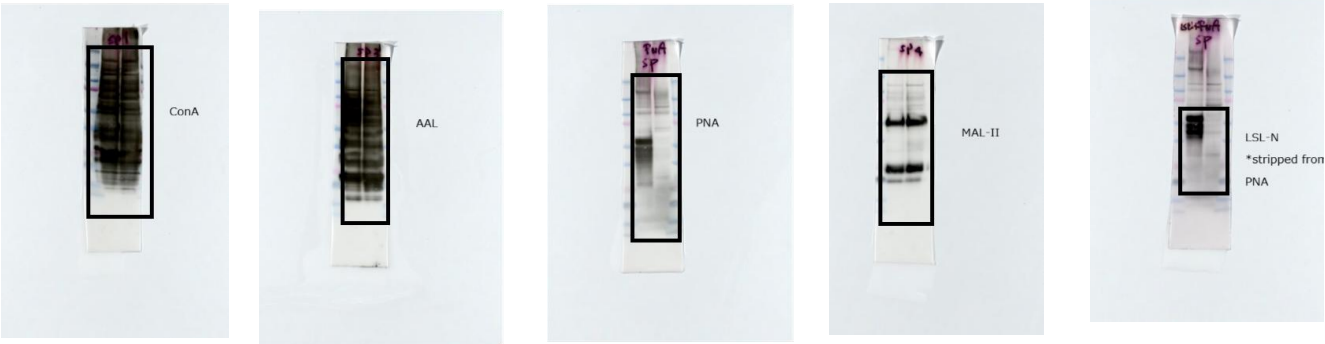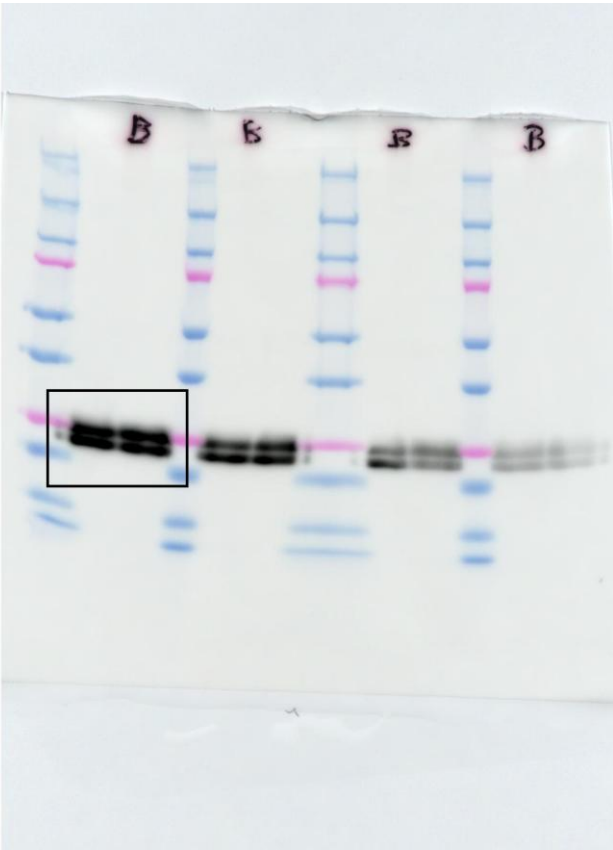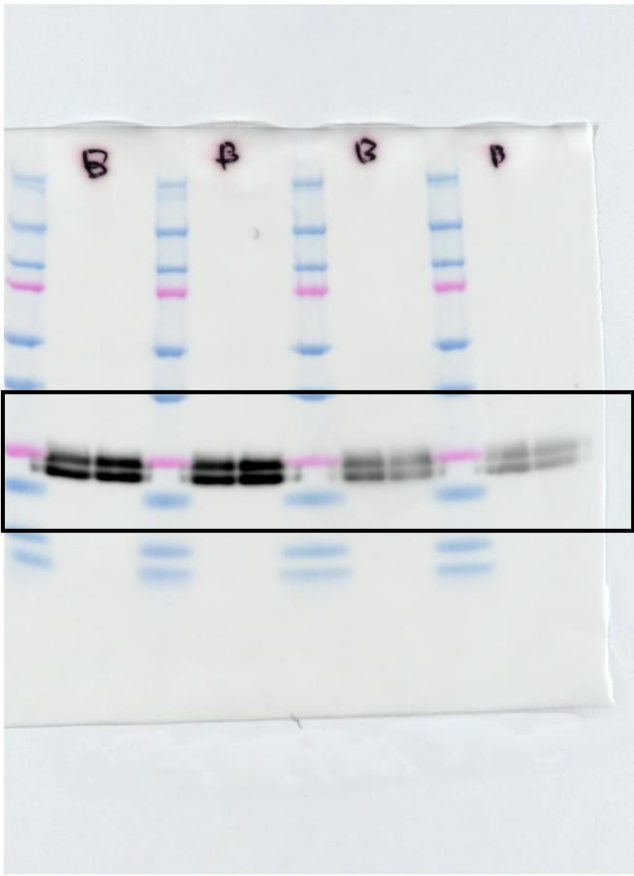

C

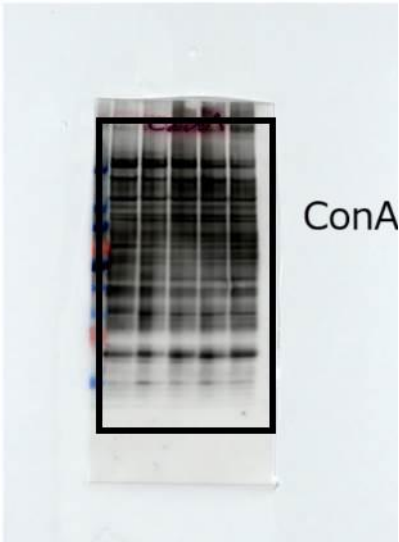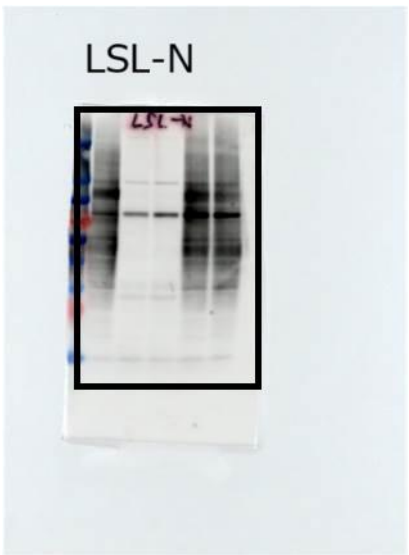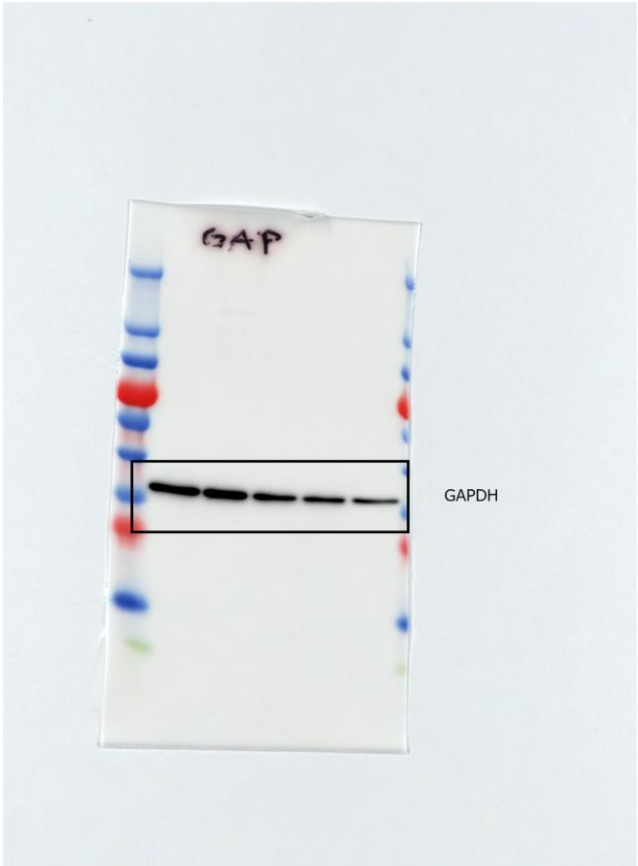

D

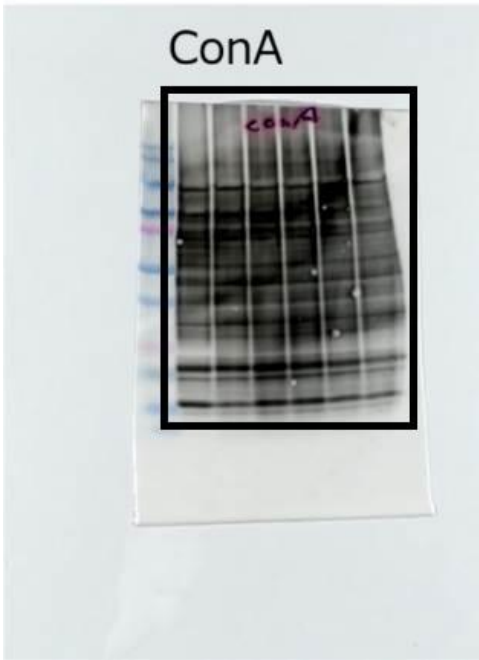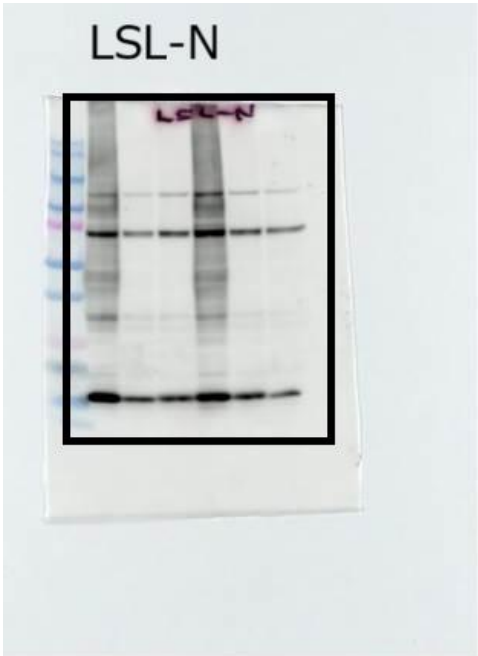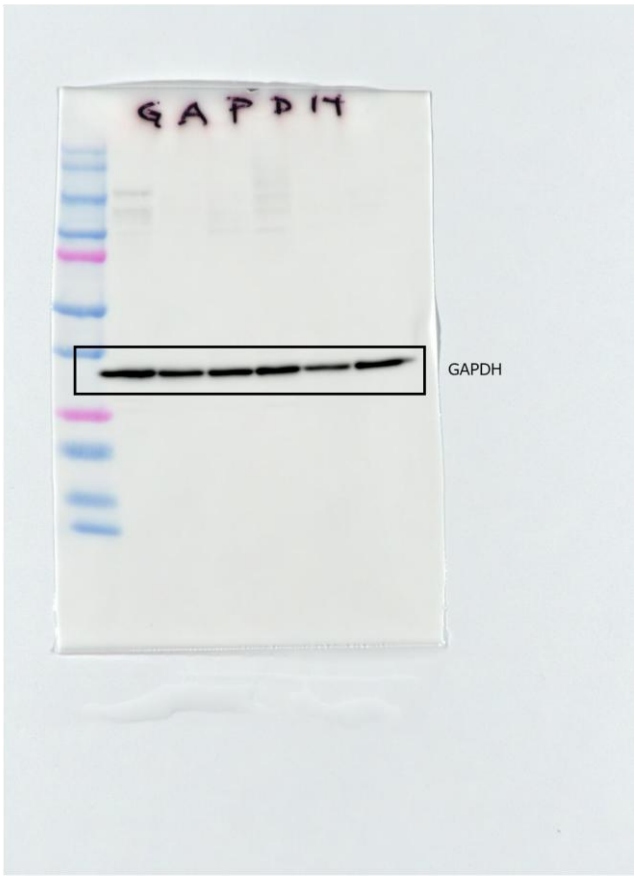

Supplement: Figure 7—source data 1. [file elife-107494-fig7-data1.pdf]

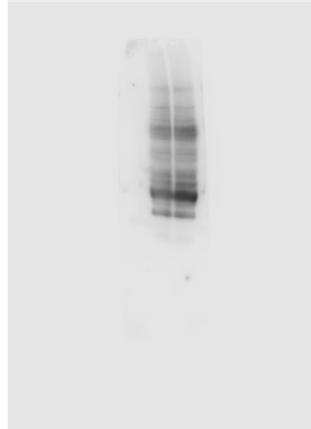

Supplement: Figure 7—source data 2. [file elife-107494-fig7-data2.zip › Figure7-source data2/f7a-AAL.tif]

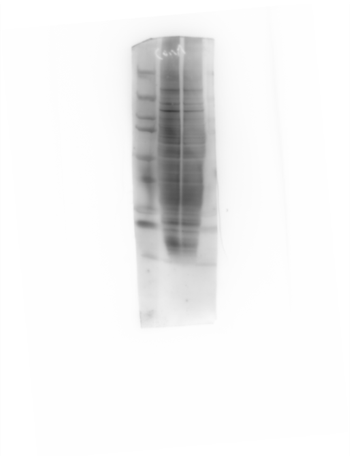

Supplement: Figure 7—source data 2. [file elife-107494-fig7-data2.zip › Figure7-source data2/f7a-ConA.tif]

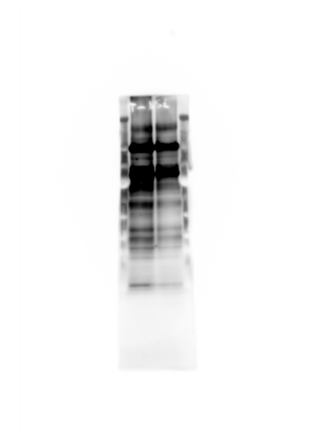

Supplement: Figure 7—source data 2. [file elife-107494-fig7-data2.zip › Figure7-source data2/f7a-LSL-N.tif]

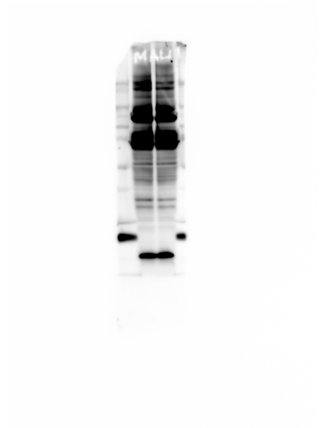

Supplement: Figure 7—source data 2. [file elife-107494-fig7-data2.zip › Figure7-source data2/f7a-MAL-II.tif]

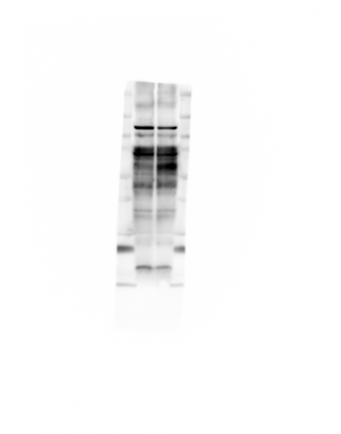

Supplement: Figure 7—source data 2. [file elife-107494-fig7-data2.zip › Figure7-source data2/f7a-PNA.tif]

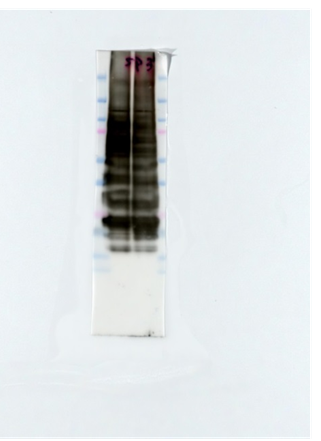

Supplement: Figure 7—source data 2. [file elife-107494-fig7-data2.zip › Figure7-source data2/f7b-AAL.tif]

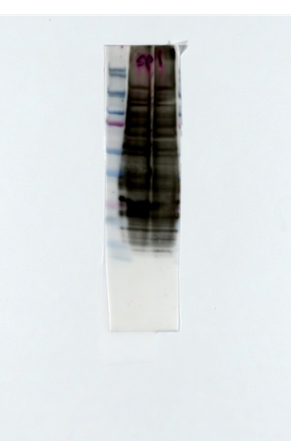

Supplement: Figure 7—source data 2. [file elife-107494-fig7-data2.zip › Figure7-source data2/f7b-ConA.tif]

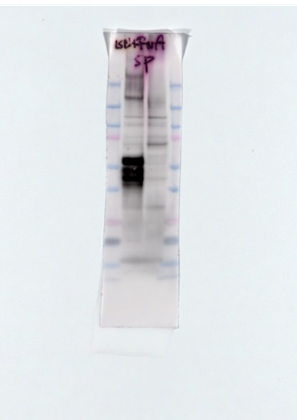

Supplement: Figure 7—source data 2. [file elife-107494-fig7-data2.zip › Figure7-source data2/f7b-LSL-N_stripped from PNA.tif]

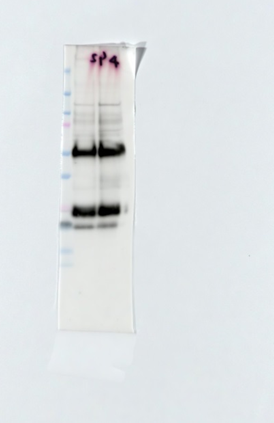

Supplement: Figure 7—source data 2. [file elife-107494-fig7-data2.zip › Figure7-source data2/f7b-MAL-II.tif]

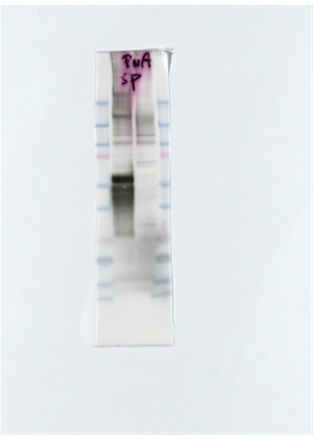

Supplement: Figure 7—source data 2. [file elife-107494-fig7-data2.zip › Figure7-source data2/f7b-PNA.tif]

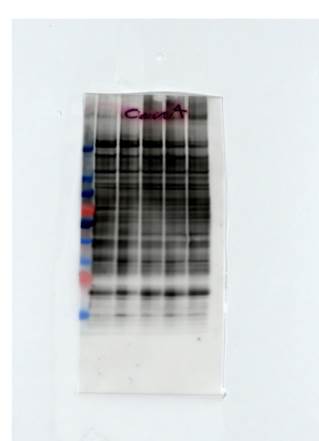

Supplement: Figure 7—source data 2. [file elife-107494-fig7-data2.zip › Figure7-source data2/f7c-ConA.jpg]

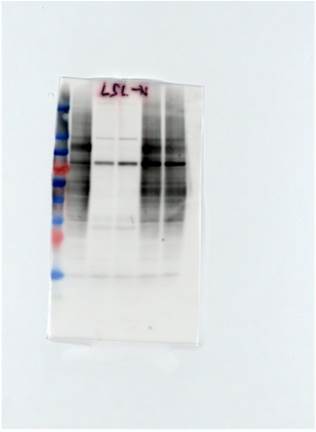

Supplement: Figure 7—source data 2. [file elife-107494-fig7-data2.zip › Figure7-source data2/f7c-LSL-N.jpg]

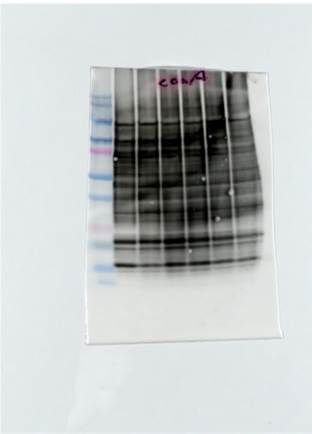

Supplement: Figure 7—source data 2. [file elife-107494-fig7-data2.zip › Figure7-source data2/f7d-ConA.jpg]

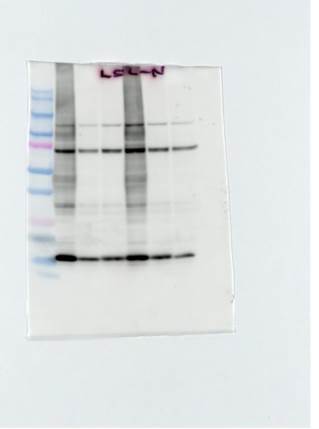

Supplement: Figure 7—source data 2. [file elife-107494-fig7-data2.zip › Figure7-source data2/f7d-LSL-N.jpg]

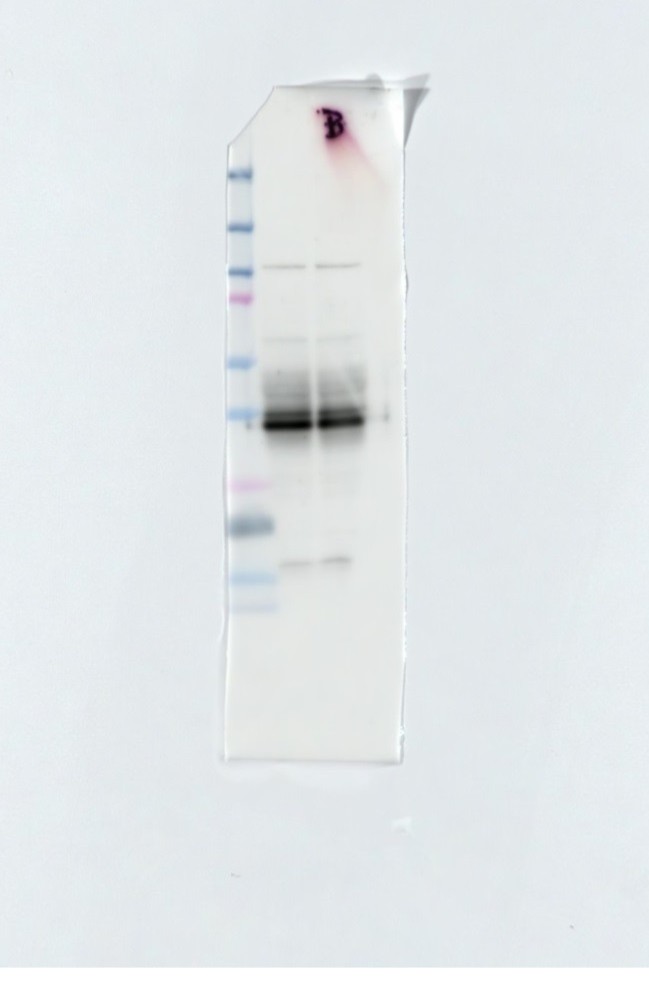

Supplement: Figure 7—source data 2. [file elife-107494-fig7-data2.zip › Figure7-source data2/Fig7a-aal-BASIGIN.jpg]

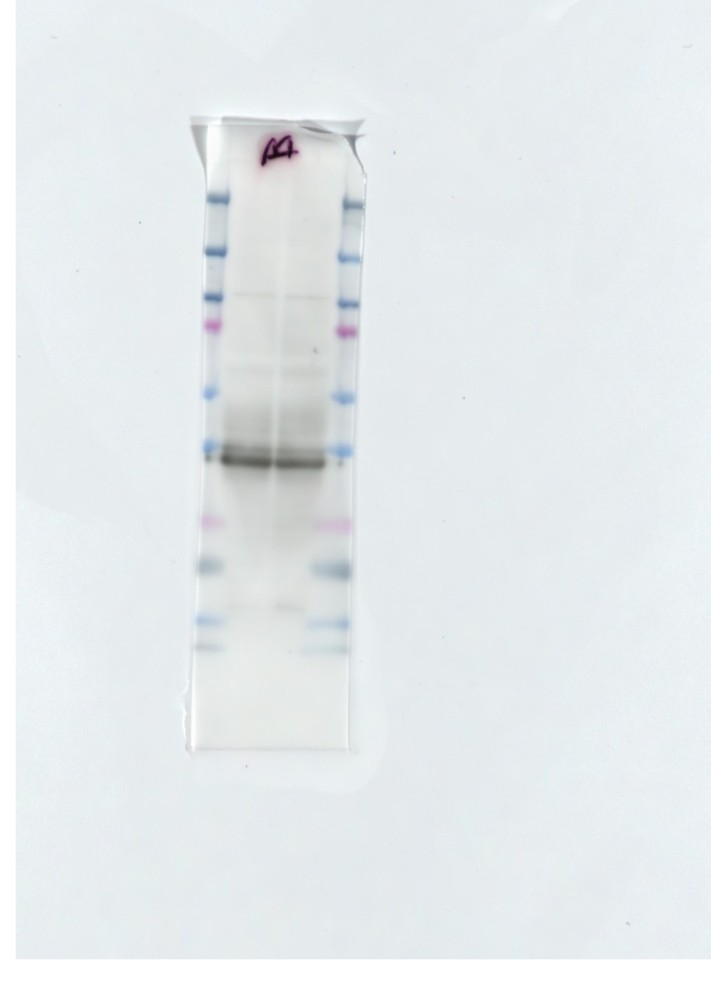

Supplement: Figure 7—source data 2. [file elife-107494-fig7-data2.zip › Figure7-source data2/fig7a-conA-BASIGIN.jpg]

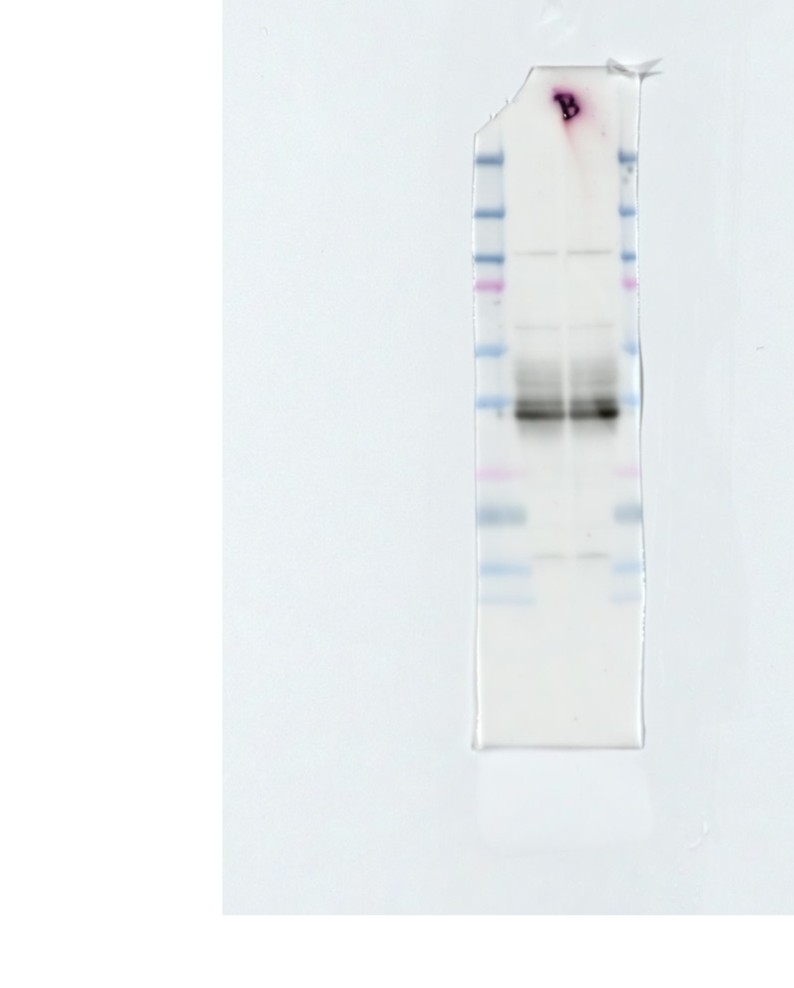

Supplement: Figure 7—source data 2. [file elife-107494-fig7-data2.zip › Figure7-source data2/Fig7a-lsl-n-BASIGIN.jpg]

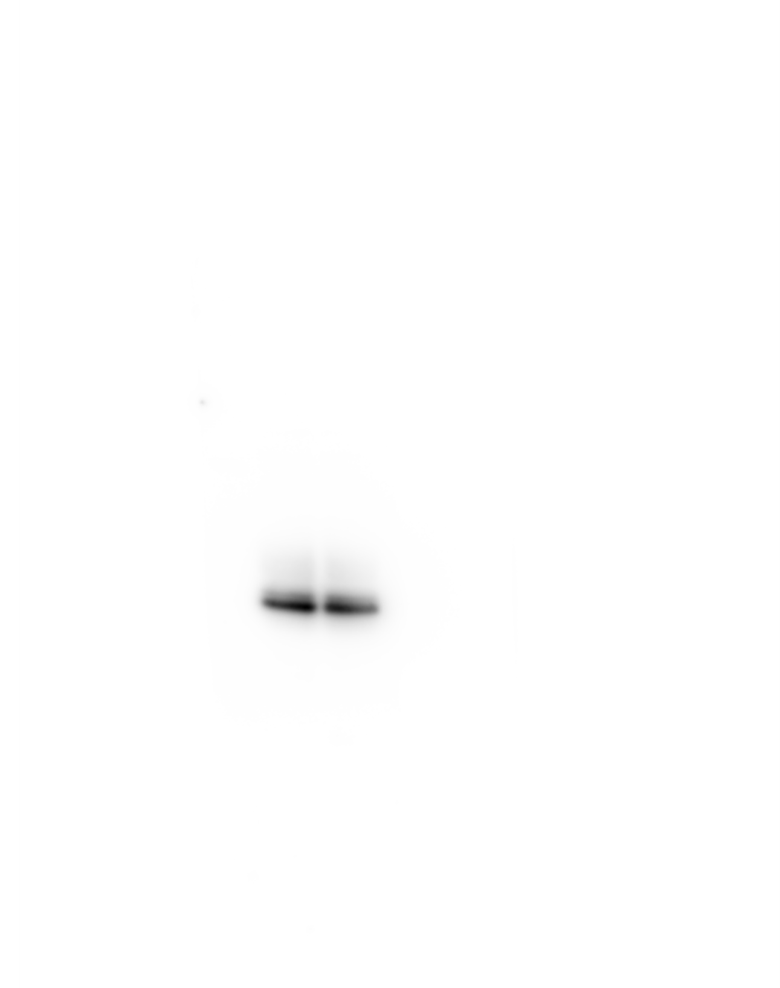

Supplement: Figure 7—source data 2. [file elife-107494-fig7-data2.zip › Figure7-source data2/Fig7a-mal-ii-BASIGIN.tif]

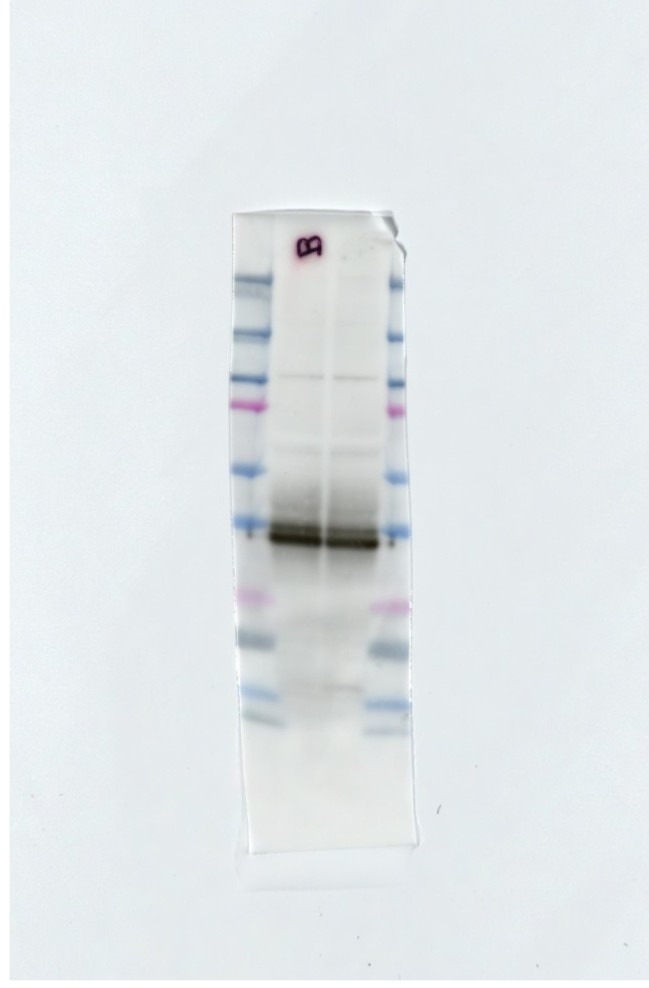

Supplement: Figure 7—source data 2. [file elife-107494-fig7-data2.zip › Figure7-source data2/Fig7a-PNA-BASIGIN.jpg]

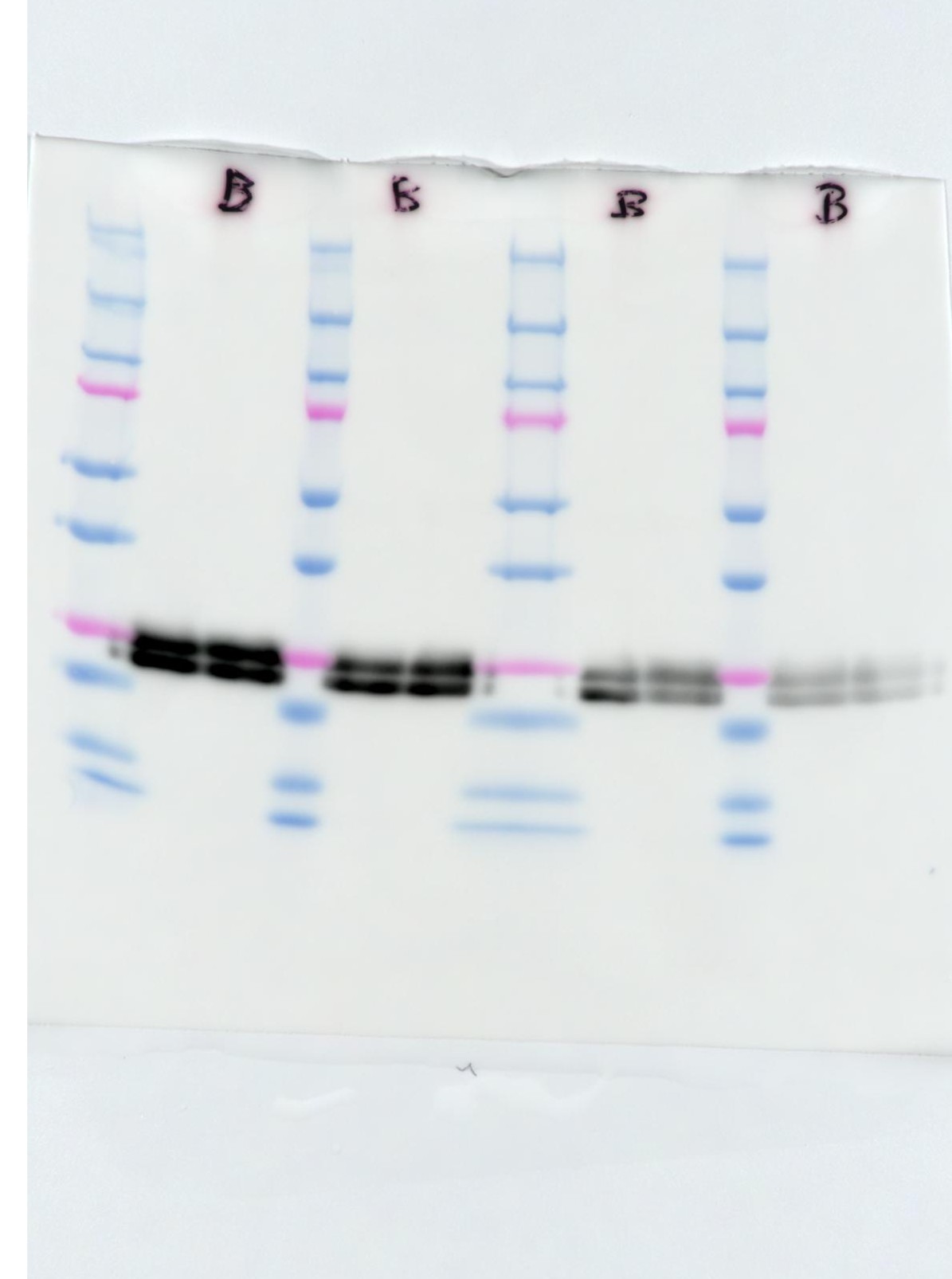

Supplement: Figure 7—source data 2. [file elife-107494-fig7-data2.zip › Figure7-source data2/Fig7b-conA-BASIGIN1.jpg]

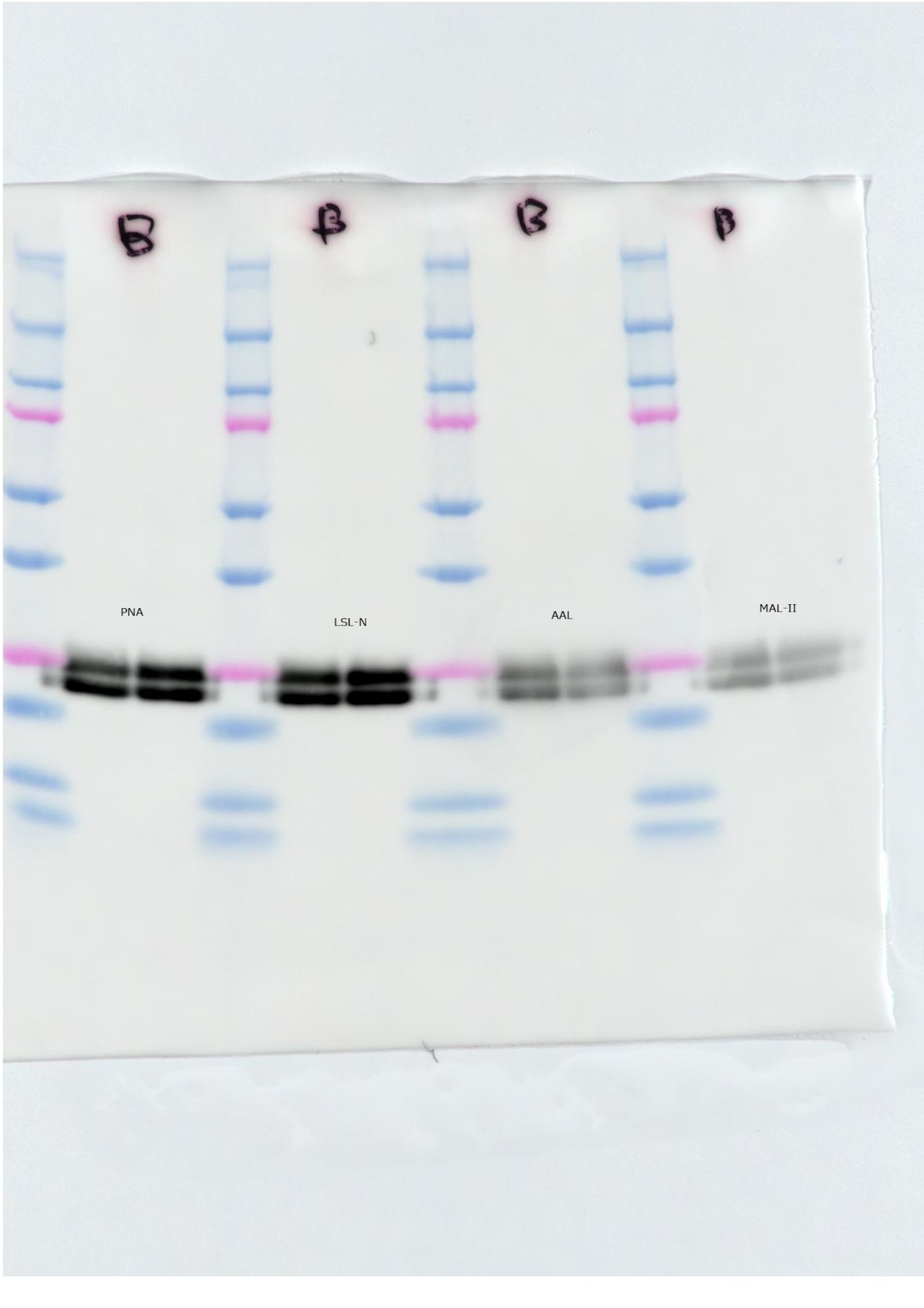

Supplement: Figure 7—source data 2. [file elife-107494-fig7-data2.zip › Figure7-source data2/Fig7b-PNA1-LSLN2-AAL3-MALII4-BASIGIN - marked.jpg]

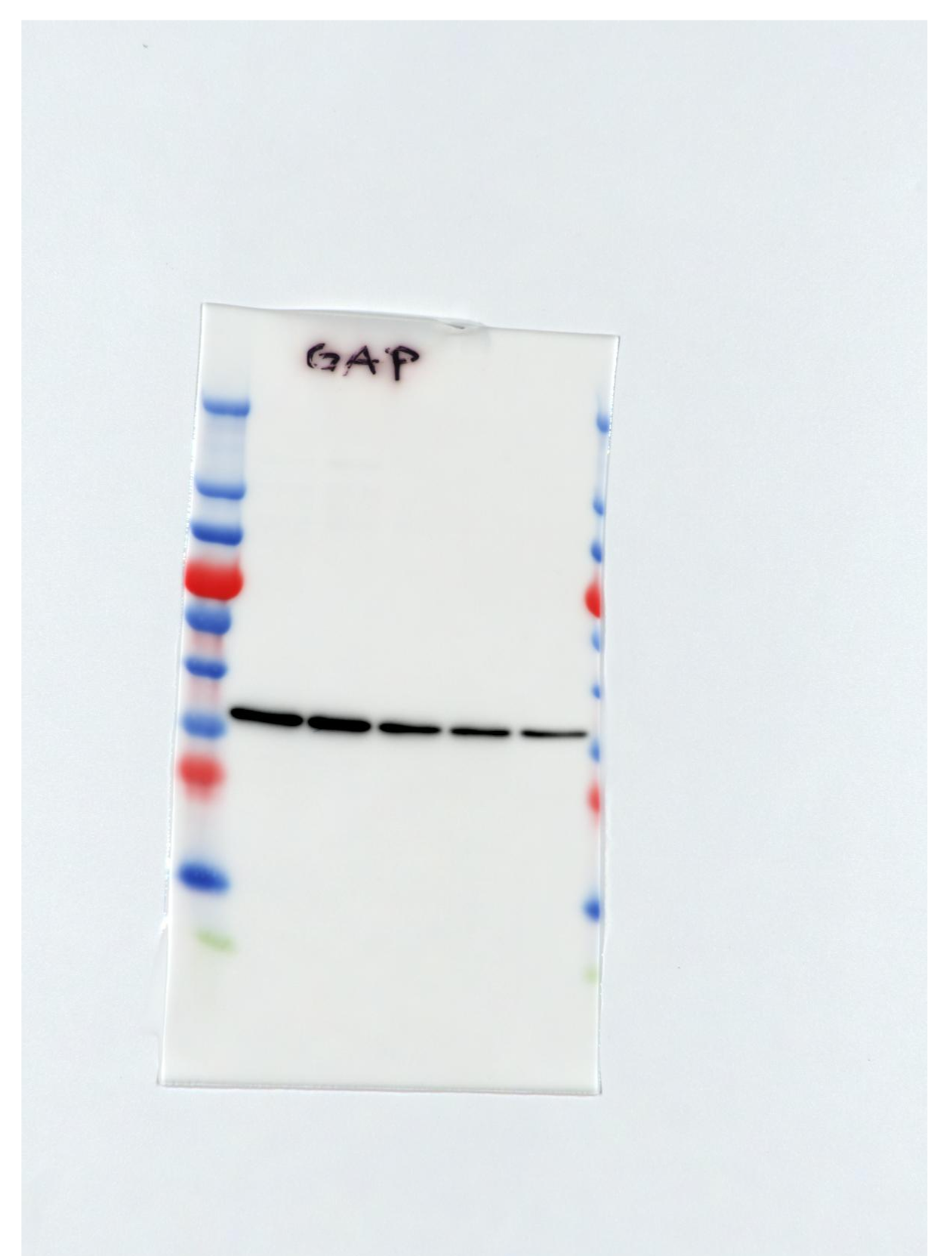

Supplement: Figure 7—source data 2. [file elife-107494-fig7-data2.zip › Figure7-source data2/Fig7C-gapdh.tif]

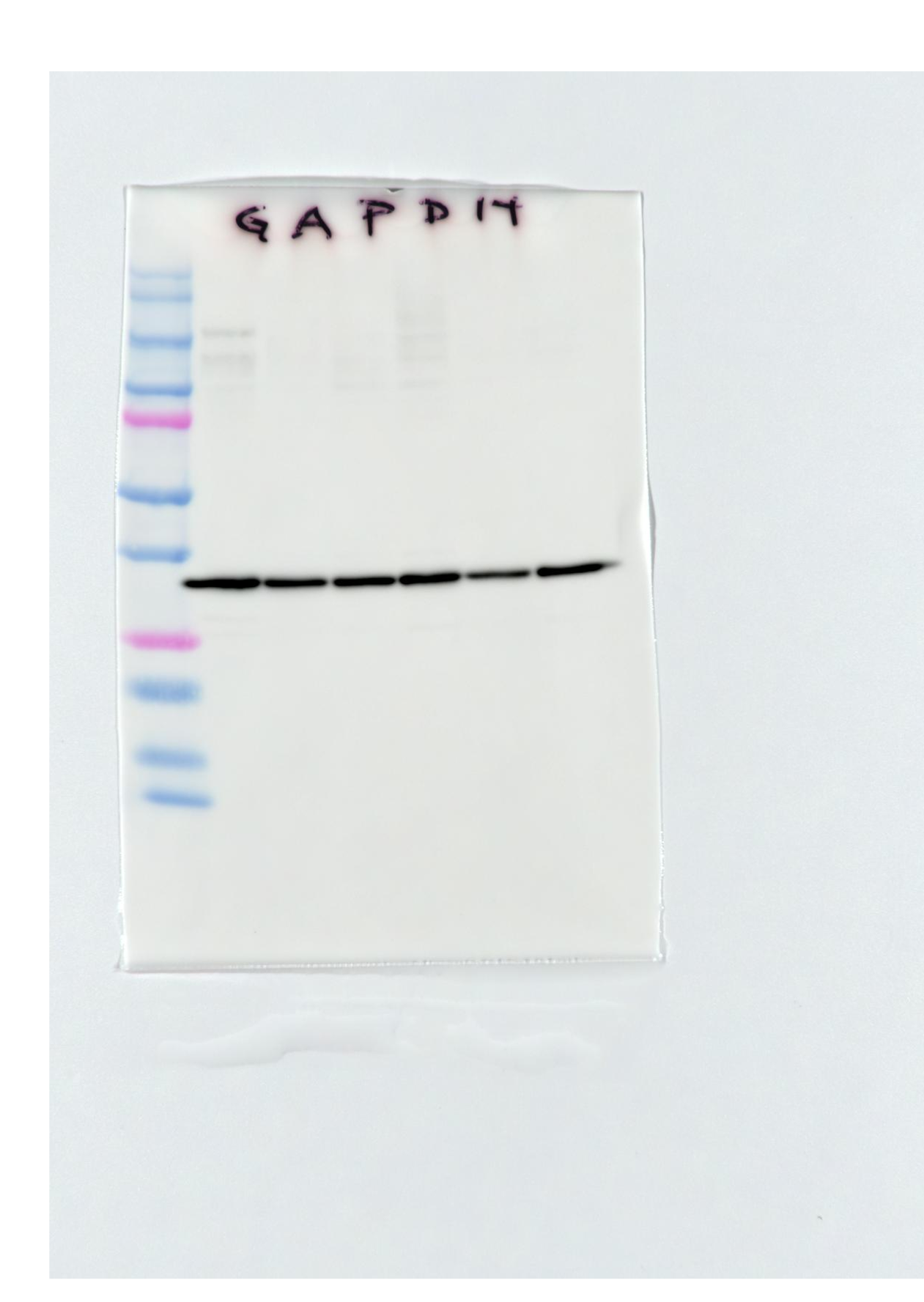

Supplement: Figure 7—source data 2. [file elife-107494-fig7-data2.zip › Figure7-source data2/fig7d-gapdh.tif]
